# High Basolateral Glucose Increases Sodium-Glucose Cotransporter 2 and Reduces Sirtuin-1 in Renal Tubules through Glucose Transporter-2 Detection

**DOI:** 10.1038/s41598-018-25054-y

**Published:** 2018-05-01

**Authors:** Hiroyuki Umino, Kazuhiro Hasegawa, Hitoshi Minakuchi, Hirokazu Muraoka, Takahisa Kawaguchi, Takeshi Kanda, Hirobumi Tokuyama, Shu Wakino, Hiroshi Itoh

**Affiliations:** Department of Internal Medicine, School of Medicine, Keio University, Tokyo, 160-8584 Japan

## Abstract

Under diabetic conditions, sodium–glucose cotransporter 2 (SGLT2) for glucose uptake in proximal tubules (PTs) increases, whereas NAD^+^-dependent protein deacetylase silent mating type information regulation 2 homolog 1 (Sirtuin-1; SIRT1) for PT survival decreases. Therefore, we hypothesized that increased glucose influx by SGLT2 reduces SIRT1 expression. To test this hypothesis, *db*/*db* mice with diabetes and high-glucose (HG)-cultured porcine PT LLC-PK1 cells in a two-chamber system were treated with the SGLT2 inhibitor canagliflozin. We also examined SIRT1 and SGLT2 expression in human kidney biopsies. In *db*/*db* mice, SGLT2 expression increased with concomitant decreases in SIRT1, but was inhibited by canagliflozin. For determination of the polarity of SGLT2 and SIRT1 expression, LLC-PK1 cells were seeded into Transwell chambers (pore size, 0.4 µm; Becton Dickinson, Oxford, UK). HG medium was added to either or to both of the upper and lower chambers, which corresponded to the apical and basolateral sides of the cells, respectively. In this system, the lower chamber with HG showed increased SGLT2 and decreased SIRT1 expression. Canagliflozin reversed HG-induced SIRT1 downregulation. Gene silencing and inhibitors for glucose transporter 2 (GLUT2) blocked HG-induced SGLT2 expression upregulation. Gene silencing for the hepatic nuclear factor-1α (HNF-1α), whose nuclear translocation was enhanced by HG, blocked HG-induced SGLT2 expression upregulation. Similarly, gene silencing for importin-α1, a chaperone protein bound to GLUT2, blocked HG-induced HNF-1α nuclear translocation and SGLT2 expression upregulation. In human kidney, SIRT1 immunostaining was negatively correlated with SGLT2 immunostaining. Thus, under diabetic conditions, SIRT1 expression in PTs was downregulated by an increase in SGLT2 expression, which was stimulated by basolateral HG through activation of the GLUT2/importin-α1/HNF-1α pathway.

## Introduction

Diabetic nephropathy (DN) is a complication of diabetes mellitus that causes end-stage renal disease^1^. Pathological manifestations of DN include glomerular and tubulointerstitial changes^2^. Tubules often represent the primary site for changes in DN^3^. Reduction in silent mating type information regulation 2 homolog 1 (Sirtuin-1; SIRT1) expression in proximal tubules (PTs) precedes that in podocytes, which constitutes an early event in patients with diabetes^4^. This downregulation of SIRT1 expression in PTs may be a prodrome of glomerular damages in DN. Nicotinamide mononucleotide, a SIRT1-related metabolite of nicotinic acid metabolism^5^, levels are reduced because of decreased SIRT1 levels in PTs, which damage podocytes. We describe this disease propagation process as “tubule-podocyte interplay.” We have previously reported that high-glucose (HG) medium reduced SIRT1 levels in cultured PTs^4^. The underlying mechanisms by which HG conditions regulate SIRT1 expression in PTs in diabetes remain unclear.

Sodium–glucose co-transporters (SGLT) mediate glucose reabsorption and cellular glucose entry. SGLTs are expressed on the apical site of PTs, with upregulated expression in db/db mice^6^. The sodium–glucose cotransporter (SGLT) comprises two isoforms: SGLT1 and SGLT2. The SGLT2 transporter mediates 90% renal glucose reabsorption and the remaining 10% occurs through SGLT1. Although SGLT2 plays a dominant role in glucose transport in PTs, SGLT1 also plays a significant role under the condition of SGLT2 inhibition or diabetic milieu. However, because SGLT1 is present in the intestinal and renal tissues, inhibition of this transporter has the potential to induce osmotic diarrhea^7^. Therefore, SGLT2 inhibitors were recently made available for clinical use as glucose-lowering reagents. Accumulating evidence suggests their protective effects on diabetic PT cells. In diabetes, SGLT2 inhibitors prevented increases in reactive oxidative species (ROS), causing apoptotic damage to PT cells^8^. Inhibition of SGLT2 restored decreased SIRT1 levels in diabetic PTs, preventing cellular senescence of PTs^9^. Therefore, we surmised that activation of SGLT2 reduces SIRT1 expression by inducing excessive glucose entry into PTs under diabetic conditions.

To elucidate a more detailed mechanism for the relationship between SGLT2 and SIRT1, we used obese-type db/db mice with diabetes treated with the SGLT2 inhibitor canagliflozin (Cana) and investigated SIRT1 and SGLT2 expressions. We also investigated the mechanism whereby HG regulates SGLT2 and SIRT1 expression in PT cells. Subsequently, we demonstrated the relationship between SGLT2 and SIRT1 expression in human kidney biopsy samples. SGLT2 inhibition may reverse reductions in SIRT1 in PT in DN, thereby providing a tissue-protective effect in DN.

## Methods

### Animal experiments

All mice were bred with a C57BL/6 genetic background. We purchased 7-week-old male *db*/*db* mice with diabetes (BKS.Cg-*Lepr*^*db*^/*Lepr*^*db*^) and *db*/*m* mice without diabetes (BKS.Cg-*Lepr*^*db*^/^+^) from CLEA Japan (Tokyo, Japan). All procedures were conducted in accordance with relevant guidelines and regulations; all protocols were approved by Keio University Animal Care and Use Committees. Throughout the study, mice were housed in individual cages and given water *ad libitum*. From arrival to use, the mice were fed a laboratory chow diet (CE-2 pellet; CLEA Japan, Inc; with the following composition: moisture, 9.3%; crude protein, 25.1%; crude fat, 4.8%; crude fiber, 4.2%; nitrogen-free extract, 50.0%; and crude ash, 6.7%). The animal room was maintained under controlled conditions (20 °C, 65% humidity, and a 12-h light/12-h dark photoperiod with lights on at 8:00 a.m.). Cana was provided by the Medicinal Chemistry Laboratory of Mitsubishi Tanabe Pharma Corporation (Osaka, Japan). Each group was given Cana mixed with their diet (powdered CE-2), which was provided *ad libitum*. The average daily dose of the drug (calculated from food intake and body weight) was as follows: Cana 0.02% w/w food admixture = 30.0 mg/kg and Cana 0.005% w/w food admixture = 7.5 mg/kg. Consistently, a recent study utilized Cana in *in vivo* settings at concentrations of 0.005%, 0.01%, and 0.03%^10–13^. Cana was administered to *db*/*db* mice (*n* = 8) for 8 weeks, beginning at 8 weeks or age. Powdered CE-2 was given to *db*/*db* mice (*n* = 8) and *db*/*m* mice (*n* = 8). At 16 weeks of age, the mice were anesthetized by pentobarbital injection (50 mg/kg) and exsanguinated through an incision in the cervical artery, under anesthesia. The kidneys were removed, weighed, and processed, as described previously^14^. The body weight of each mouse was measured every week. Plasma and urinary glucose levels were measured every 4 weeks.

### Histopathological analysis

Immunohistochemistry, using specific antibodies, was performed as detailed in the Supplementary Materials.

### Cell culture

We analyzed polarity of SGLT2 expression using LLC-PK1 porcine renal epithelial cells (ATCC, Manassas, VA, MD, USA; lot number: 59681631). The culture cells were cultivated at 37 °C, 5% CO_2,_ in Dulbecco’s modified minimal essential medium supplemented with heat-inactivated 10% fetal bovine serum (Thermo Fisher Scientific, San Jose, CA, USA). LLC-PK1 cells at passage 8^th^–30^th^ cells were used. We determined the effects of D-glucose on polarity of SGLT2 expression by seeding cells onto porous tissue culture inserts (pore size, 0.4 µm; Becton Dickinson, Oxford, UK). All experiments were performed using confluent monolayers of cells under serum-free conditions following growth arrest in serum-free medium. We used Cana at 100 and 500 nM, which effectively and selectively blocked SGLT2 expression without significantly inhibiting SGLT1 expression^15^. We determined these concentrations based on previous pharmacokinetics data^16^. In that study, when Cana was administered to patients with diabetes at a dosage of 100 mg/day, maximum serum concentrations reached 1126 ng/ml (2533 nM), equivalent to 43 nM, given that the protein-binding ratio of Cana was 98.3%. These cells were used for the immunofluorescence analysis of SGLT2 expression, incorporation of 2-[N-(7-nitrobenz-2-oxa-1,3-diazol-4-yl)amino]-2-deoxy- D-glucose (2-NBDG), immunoprecipitation analysis, and analysis of the effect of small interfering RNAs (siRNAs) on importin expression. The cells were cultured with 5.5 mmol/L glucose (normal glucose), 22.5 mmol/L glucose (high glucose), or 5.5 mmol/L glucose plus 17.0 mmol/L mannitol (mannitol); these concentrations were within the normal range that is relevant for humans with diabetes. Detailed methods are described in the Supplementary Materials.

### Extraction of the nuclear fraction

Nuclear lysates were prepared using NE-PER^TM^ Nuclear and Cytoplasmic Extraction Reagents (Thermo Fisher Scientific, San Jose, CA, USA), as described previously^17^. Briefly, cells were washed twice with ice-cold PBS buffer (1 mM KH_2_PO_4_, 155 mM NaCl, and 3 mM Na_2_HPO_4_-7H_2_O) and centrifuged at 500 × *g* for 3 min. The cell pellet was suspended in 200 μl of ice-cold cytoplasmic extraction reagent I by vortexing. The suspension was incubated on ice for 10 min, followed by addition of 11 μl of the second cytoplasmic extraction reagent II. After vortexing for 5 s, the suspension was incubated on ice for 1 min and centrifuged for 5 min at 16 000  × *g*. The supernatant fraction (cytoplasmic extract) was transferred to a pre-chilled tube. The insoluble pellet fraction, which contains crude nuclei, was resuspended in 100 μl of the nuclear extraction reagent by vortexing for 15 s and incubated on ice for 10 min, then centrifuged for 10 min at 16 000  × *g*. The resulting supernatant, constituting the nuclear extract, was used for subsequent experiments.

### Immunoblotting and quantitative PCR of mouse kidney tissues and LLC-PK1 cells

Immunoblotting and real-time PCR were performed, as described previously^4^. Primer sequences and other details are described in the Supplementary Methods.

### Human renal needle-biopsy specimens

We obtained renal needle-biopsy specimens from 11 patients with DN. Before study enrollment, written informed consent was obtained in all patients. Patient clinical data were acquired at the time of renal biopsy and are summarized in Table 1. The study was performed in accordance with the Declaration of Helsinki and the study protocol was approved by the human ethics review committee of the Department of Internal Medicine, School of Medicine, Keio University. Immunohistochemical analysis of SIRT1 and SGLT2 expression for each human sample was performed, as described previously^18^. Further details are provided in the Supplementary Methods.

**Table 1:** Clinical parameters of patients with diabetic nephropathy (DN) and controls at the time of renal needle biopsy.

Sample name	Sex	Age (years)	Serum creatine (mg/dL)	Proteinuria (g/day)	eGFR (ml/min/1.73 m^2^)	HbA1c (%)	Fasting blood glucose (mg/dl)
Diabetic Nephropathy							
DN-1	male	83	1.81	1.6	28.5	6.6	191
DN-2	male	42	1.13	4.9	58.1	6.3	139
DN-3	male	62	1.73	1.5	32.6	6.1	97
DN-4	male	47	1.82	5.4	33.4	6.1	98
DN-5	male	80	1.23	10.2	44.0	6.0	97
DN-6	male	56	2.93	12.0	18.9	6.4	144
DN-7	male	75	1.57	1.4	33.6	6.1	205
DN-8	male	59	1.88	0.5	71.2	5.8	136
DN-9	female	64	1.89	2.9	36.3	6.1	167
DN-10	female	51	0.54	4.6	91.0	5.9	135
DN-11	male	61	0.69	7.7	89.0	7.2	155
Controls							
CO-1	female	39	0.61	<0.1	86.2	5.2	84
CO-2	male	27	0.87	<0.1	88.1	4.9	99
CO-3	female	51	0.64	<0.1	76.7	5.3	101
CO-4	female	35	0.66	<0.1	84.9	5.0	75
CO-5	male	42	0.83	<0.1	81.4	5.5	91

### Statistical analysis

Data are expressed as mean ± standard deviation. Differences were evaluated using one-way ANOVA, followed by Bonferroni’s multiple-comparison *post hoc* test (GraphPad Prism, version 4.0, GraphPad Software, La Jolla, CA, USA). The criterion for statistical significance was a *P* value < 0.05.

## Results

### Effects of canagliflozin treatment in *db*/*db* mice

**Figure 1:**
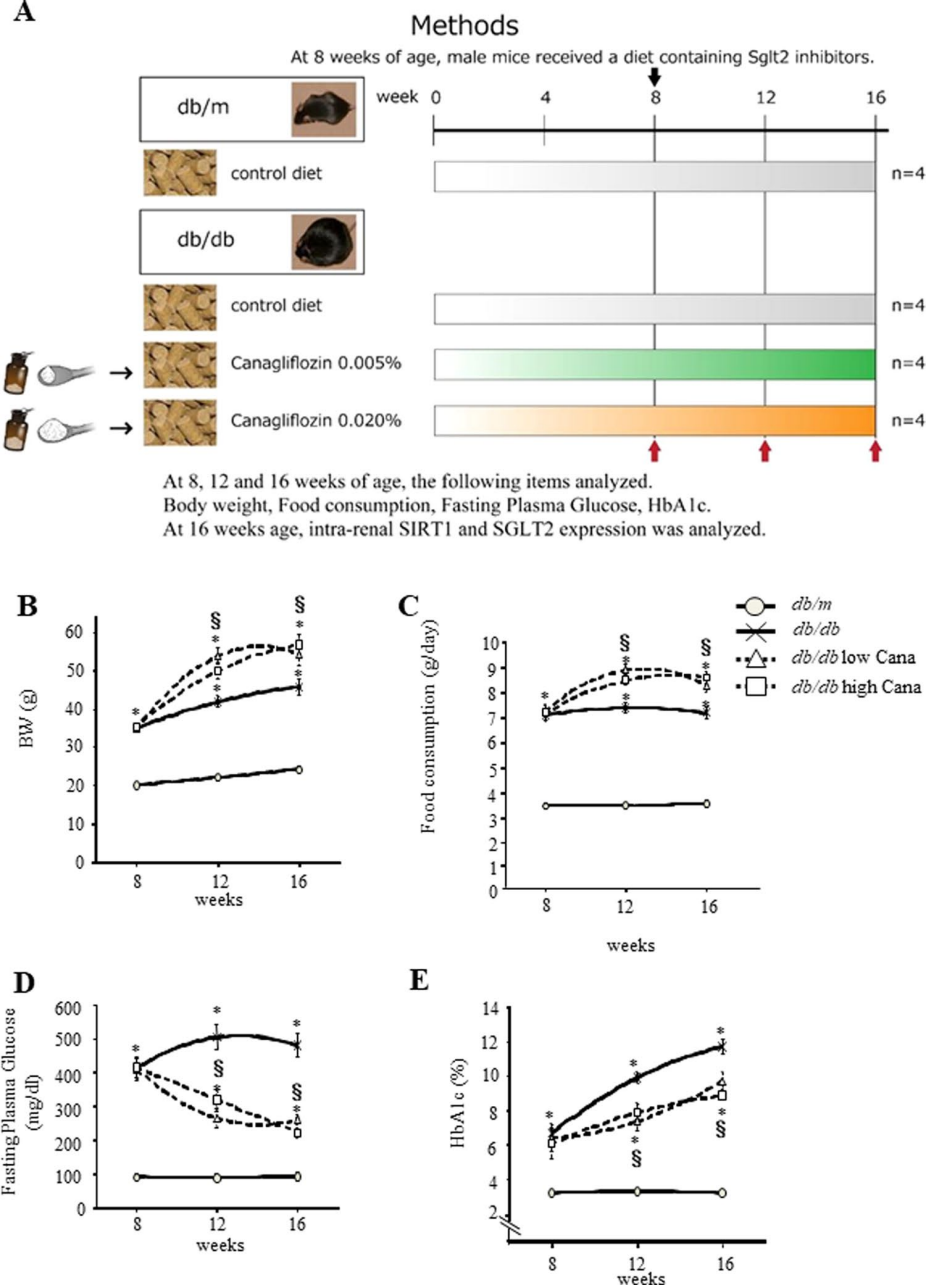
Effect of canagliflozin on parameters of glucose metabolism. (**A**) Schedule for canagliflozin (Cana) treatment and experimental groups. Effects of Cana on body weight (**B**), food intake (**C**), fasting plasma glucose level (**D**) and glycated Hb levels (**E**) in *db*/*db* mice. HbA1c, hemoglobin A1c. Data represent the mean ± SEM (*n* = 8 mice/group). *P < 0.05 vs. *db*/*m* and ^§^*P* < 0.05 vs. *db*/*db*.

To determine the effect of Cana in *db*/*db* mice, we measured physiological and metabolic parameters of four groups of mice (Fig. 1A). Mean body weights of the *db*/*db* groups were higher than those of the *db*/*m* group. Moreover, the mean body weight of the *db*/*db* group treated with 7.5 or 30.0 mg/kg/day Cana (*db*/*db* + 7.5 mg Cana group and *db*/*db* + 30.0 mg Cana group, respectively) was higher than that of the *db*/*db* group (Fig. 1B). Furthermore, daily food consumption of the Cana groups was greater than that of the *db*/*m* mice (Fig. 1C). Plasma fasting glucose levels and HbA1c increased in the *db*/*db* mice, although Cana significantly reduced these values compared with those of the *db*/*db* mice (Fig. 1D and E, respectively).

### Effects of Cana on renal SGLT2 expression

**Figure 2:**
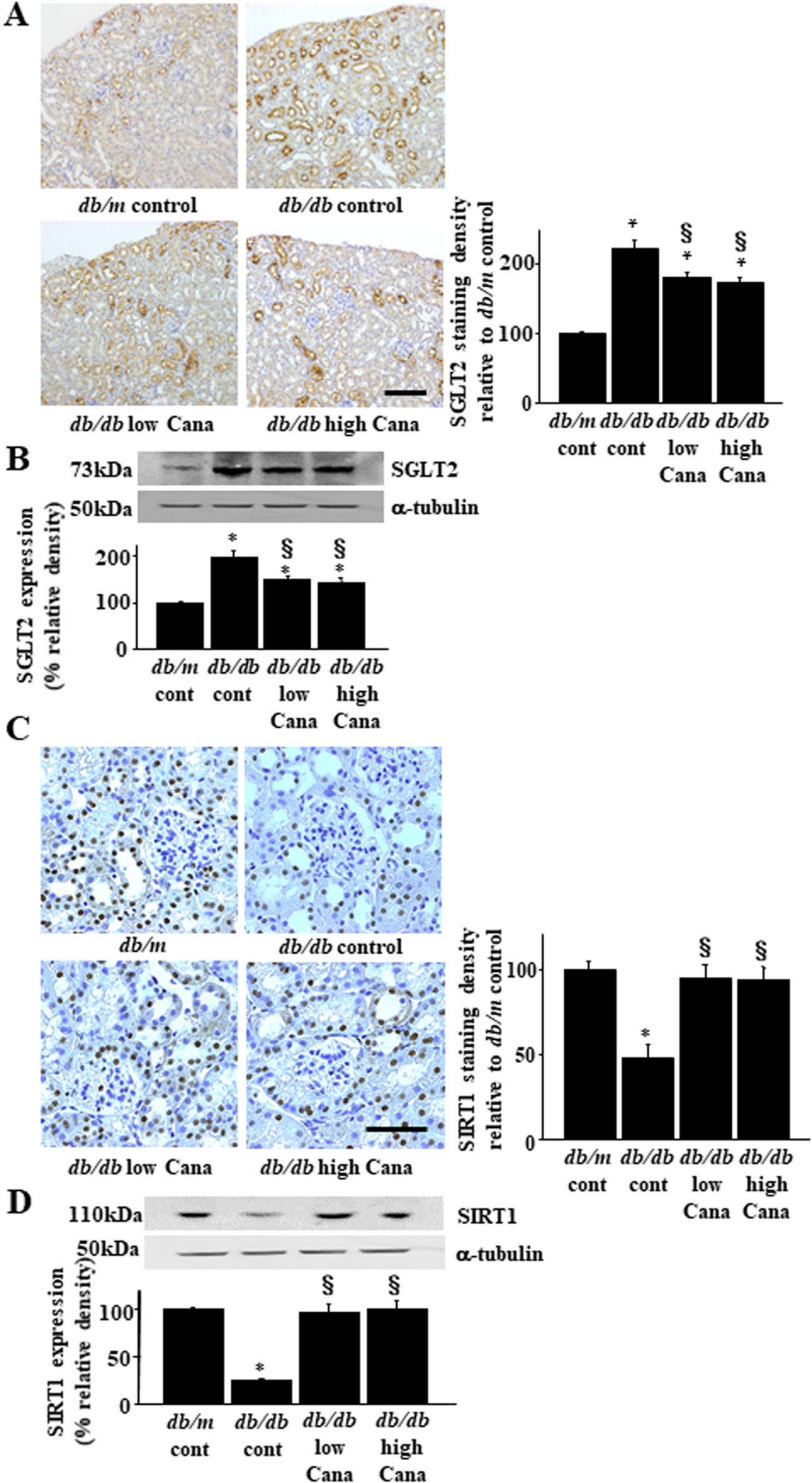
Effect of Cana on SGLT2 and SIRT1 expression in *db*/*db* mice. (**A**) Immunohistochemical analysis using a SGLT2-specific antibody. Representative kidney sections are shown for each group of mice. Quantitation of relative density is shown in the bar graph in the right panel. Scale bar, 100 µm. *P < 0.05 vs. *db*/*m* control and ^§^*P* < 0.05 vs. *db*/*db* control (n = 8 mice/group) (**B**) The upper panel shows representative immunoblotting analysis of SGLT2 expression. The Bar graph in the lower panel indicates quantification of SGLT2 levels. Protein expression was normalized to that of α-tubulin. Relative protein levels are shown as the fold-change to the *db*/*m* (control) group. *P < 0.05 vs. *db*/*m* control and ^§^*P* < 0.05 vs. *db*/*db* control. The results are representative of four independent experiments. (**C**) Immunohistochemical analysis using a SIRT1-specific antibody. Representative kidney sections are shown for each group of mice. Quantitation of relative density is shown in the bar graph. Scale bar, 50 µm. *P < 0.05 vs. *db*/*m* control and ^§^*P* < 0.05 vs. *db*/*db* control (*n* = 8 mice/group). (**D**) The upper panel shows representative immunoblotting analysis of SIRT1 expression. The bar graph in the lower panel indicates quantification of SIRT1 levels. Protein expression was normalized to that of α-tubulin. Relative protein levels are shown as the fold-change to the *db*/*m* (control) group. *P < 0.05 *db*/*m* control and ^§^*P* < 0.05 vs. *db*/*db* control. Results are representative of four independent experiments.

To determine effects of diabetes and Cana treatment on SGLT2 expression, we performed immunohistochemistry (Fig. 2A) and immunoblotting (Fig. 2B) analyses. Renal SGLT2 expression increased in *db*/*db* mice with diabetes compared with that in *db*/*m* mice, and this increase was attenuated by Cana treatment in *db*/*db* mice (Fig. 2A,B). Glucose transporter 2 (GLUT2) expression, which is expressed on the basolateral side of the PT cell, did not change in *db*/*db* mice or *db*/*db* mice treated with Cana (Supplementary Fig. 1). SIRT1 expression decreased in *db*/*db* mice with diabetes compared with that in *db*/*m* mice, which was rescued by Cana treatment in mice with diabetes (Fig. 2C,D). There was no significant difference in SGLT2 or SIRT1 expression between the *db*/*db* + 7.5 and *db*/*db* + 30.0 mg Cana groups.

### Basolateral HG stimulates SGLT2 expression

**Figure 3:**
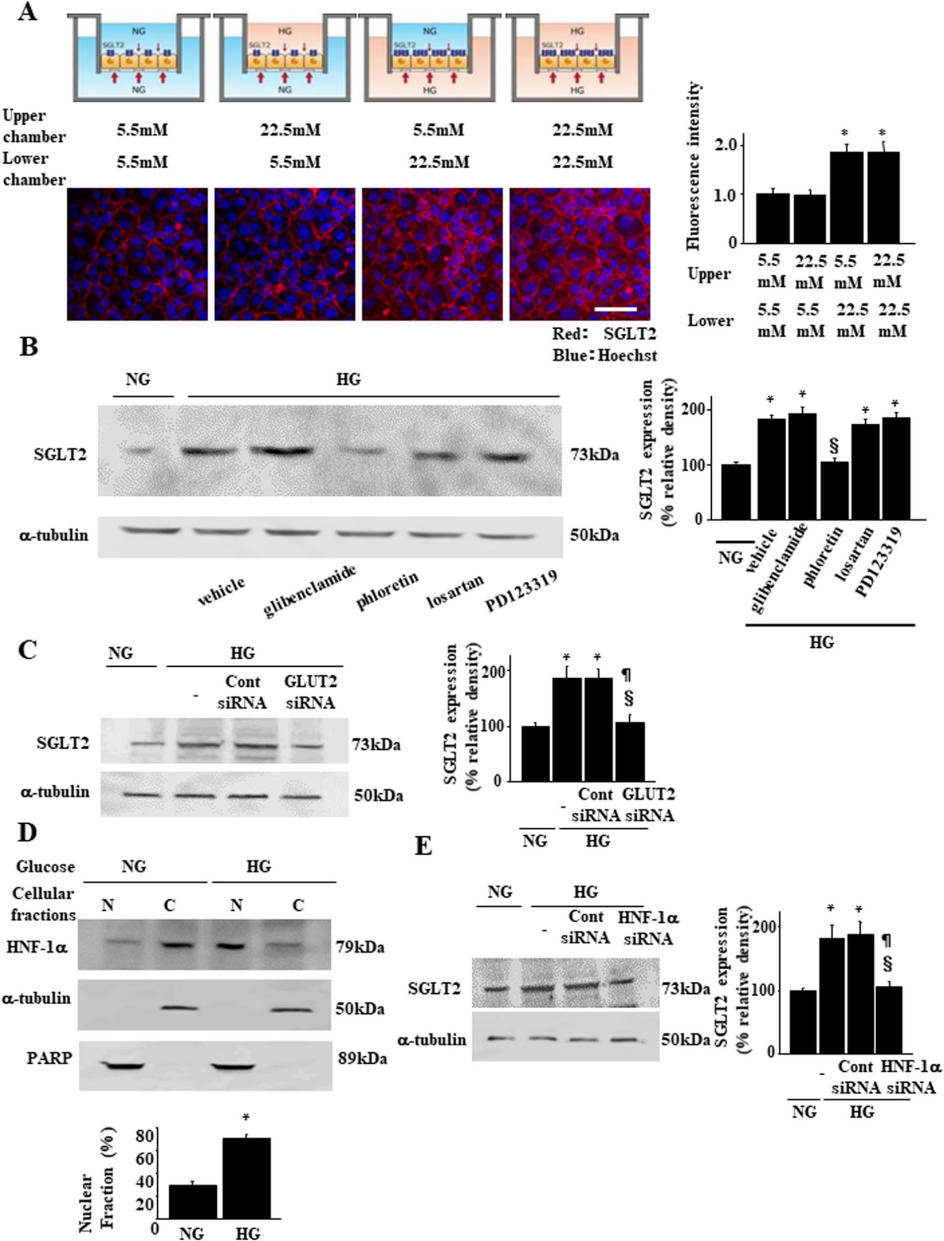
Effect of high glucose on SGLT2 expression in a two-chamber culture system. (**A**) Confluent growth-arrested cell monolayers were stimulated on the apical or basolateral side for up to 24 h, as described in the Methods section. Representative SGLT2 fluorescence of the four groups of culture conditions is shown. The bar graph represents the fluorescence intensity of each group (*P < 0.05 vs. the group with 5.5 mM D-glucose in the lower chamber and 5.5 mM D-glucose in the upper chamber, *n* = 4 independent experiments. Scale bar, 50 µm. (**B**) Effects of inhibitors for candidate signaling pathways on SGLT2 expression. LLC-PK1 cells were treated with a Na-K ATPase channel inhibitor (glibenclamide, 50 µM), GLUT2 inhibitor (phloretin, 100 µM), angiotensin II type 1 receptor (AT1R) inhibitor (losartan, 1 µM), and AT2R inhibitor (PD123319, 100 µM). Results are representative of four independent experiments. The bar graph represents the band intensity of each group (*P < 0.05 vs. NG group and ^§^*P* < 0.05 vs. HG group*, n* = 4 independent experiments). (**C**) Effects of *Glut2*-siRNA (100 nmol/L) or non-targeting control siRNA (100 nmol/L) on SGLT2 expression. Results are representative of four independent experiments. The bar graph in the right panel indicates the quantification of SGLT2 levels. Protein expression was normalized to that of α-tubulin. Relative protein levels are shown as the fold-change to the NG group. *P < 0.05 vs. NG group, ^§^*P* < 0.05 vs. HG group, and ^¶^*P* < 0.05 vs. HG with control siRNA, *n* = 4. (**D**) Subcellular fractionation and immunoblotting demonstrate HG-induced redistribution from the cytoplasm to the nucleus of HNF-1α. HG, high D-glucose (22.5 mM) condition; NG, normal D-glucose (5.5 mM) condition; N, nuclear fraction; C, cytosolic fraction. HNF-1α protein levels were determined by densitometry and are indicated for each fraction. The percent of HNF-1α in the nucleus relative to the total HNF-1α was calculated using values determined using a densitometry. *P < 0.05 vs. NG group. Results are representative of four independent experiments. (**E**) Effects of HNF-1α-siRNA (100 nmol/L) or non-targeting control siRNA (100 nmol/L) on SGLT2 expression. The bar graph in the right panel indicates quantification of SGLT2 levels. Relative protein levels are shown as the fold-change to the NG (control) group. *P < 0.05 vs. NG group, ^§^*P* < 0.05 vs. HG group, and ^¶^*P* < 0.05 vs. HG with control siRNA, *n* = 4 independent experiments.

Given that PT cells retain membrane protein polarity, we examined how ambient glucose affects SGLT2 expression using a two-chamber culture system. Confluent LLC-PK1 cells that exhibit apical and basolateral polarity^19^ were serum-starved on porous tissue culture inserts and then exposed to normal (5.5 mM) or high (22.5 mM) D-glucose in the apical or basal chamber for 24 h (Fig. 3A). SGLT2 expression increased following the addition of 22.5 mM D-glucose to the basolateral, but not following that to the apical chamber (Fig. 3A). Next, we investigated intracellular signal transduction in PTs, directed from the basolateral to the apical side, that controls SGLT2 expression. Na-K ATPase^20^, GLUT2^21^, angiotensin II type 1 receptor (AT1R)^22^, and AT2R^23^ are located within the basolateral membrane of PT cells and transduce signals elicited by HG. We added an inhibitor for each molecule, i.e., glibenclamide^24^, phloretin^25^, losartan^26^, and PD123319^27^, respectively, to the lower chamber. HG-induced increases in SGLT2 expression were inhibited by phloretin, a GLUT2 inhibitor (Fig. 3B). HG-induced SGLT2 expression upregulation was inhibited by a *Glut2*-specific siRNA (Fig. 3C).

Next, we investigated the regulation of transcription factors that control SGLT2 expression. It is possible that hepatic nuclear factor-1α (HNF-1α) plays a key role because when translocated to the nucleus, it upregulates *Sglt2* transcription^28^. HNF-1α was mainly detected in the cytoplasmic fraction of cells cultured under normal glucose concentrations (Fig. 3D). In contrast, HNF-1α translocated to the nucleus in the presence of HG (Fig. 3D), thereby suggesting that it can function as a transcription factor in HG conditions. Treatment with a specific siRNA for HNF-1α blocked HG-induced SGLT2 expression upregulation (Fig. 3E). Thus, basolateral HG upregulated SGLT2 expression by activating GLUT2 and transcription factor HNF-1α nuclear translocation.

### Importin-α1 mediates basolateral HG stimulation for SGLT2 expression

**Figure 4:**
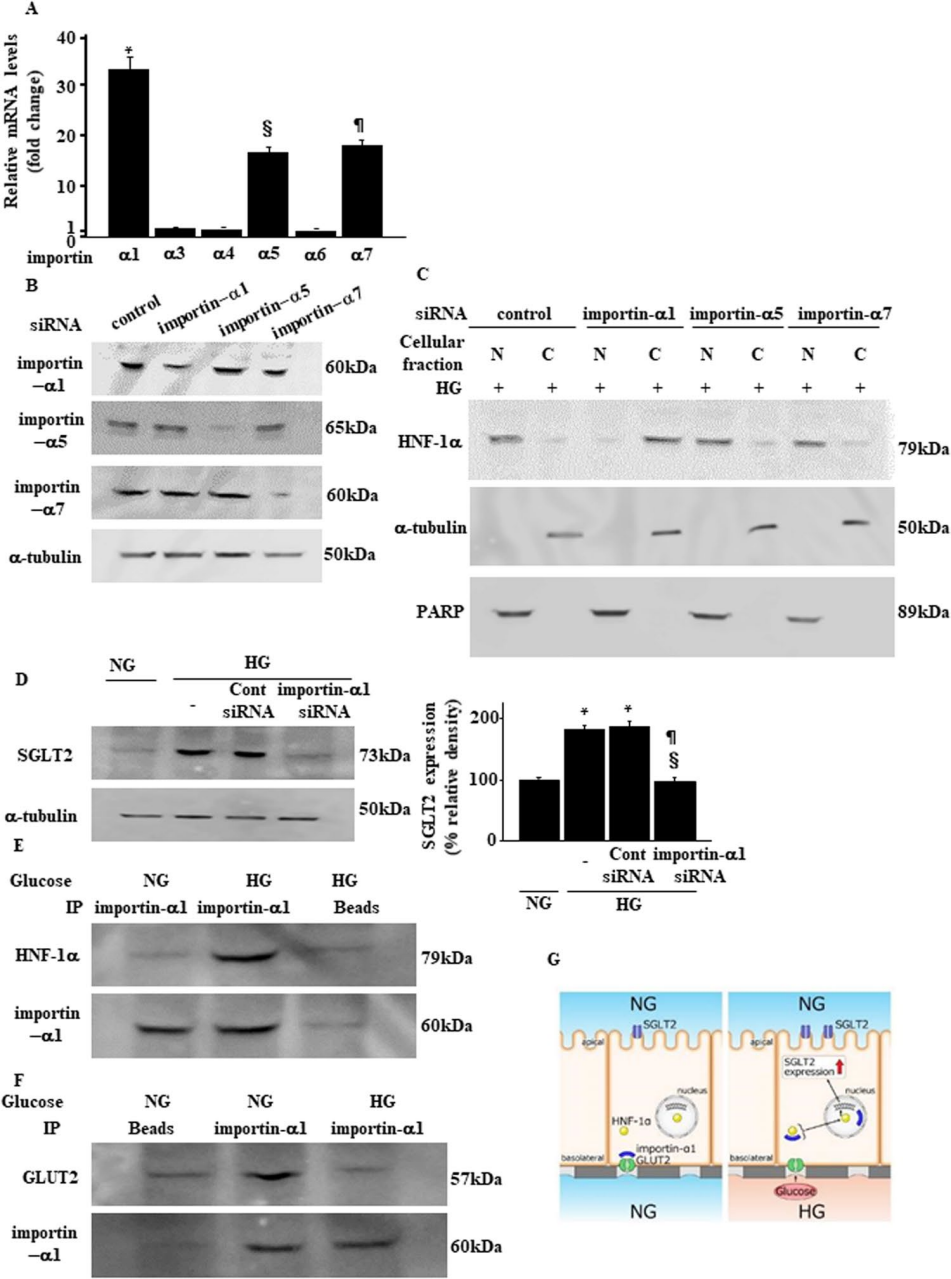
The GLUT2/importin-α1/HNF-1α pathway mediates high-glucose-induced SGLT2 expression. (**A**) Quantitative real-time PCR analysis of the relative abundance of mRNAs encoding importins in LLC-PK1 cells. Real-time PCR data were normalized to those of the *GAPDH* mRNA. Relative fold-differences were calculated using the mean value (*n* = 6) of importin-α6. (**B**) Immunoblotting confirmed the efficiency of siRNA knockdown of each of the representative importin-α isoform that was abundantly expressed in PTs. Results are representative of four independent experiments. (**C**) Subcellular fractionation and immunoblotting analysis of the localization of HNF-1α. LLC-PK1 cells were transfected with the indicated siRNA duplexes and 48 h later, cells were treated with media containing high glucose levels. Cytoplasmic and nuclear lysates were collected and analyzed using immunoblotting with an anti-HNF-1α antibody. Results are representative of four independent experiments. (**D**) LLC-PK1 cells were transiently transfected with siRNAs targeting importin-α1 or a non-targeting control siRNA. Whole cell lysates were prepared 48 h post-transfection and analyzed using immunoblotting. Results are representative of four independent experiments. The bar graph represents the band intensity of each group (*P < 0.05 vs. NG group and ^§^*P* < 0.05 vs. HG group*, n* = 4). (**E**,**F**) Immunoprecipitation of nuclear extracts from LLC-PK1 cells that were treated with NG or HG. Importin-α1 formed complexes with HNF-1α (**E**), whereas it dissociated from GLUT2 (**F**) under HG conditions. Results are representative of four independent experiments. (**G**) Model for the molecular mechanism by which the GLUT2/importin-α1/HNF-1α pathway is involved in HG-induced SGLT2 expression upregulation.

We next explored the underlying mechanism by which HG initiates GLUT2-mediated signal transduction and HNF-1α nuclear translocation. GLUT2 acts as a metabolic sensor^29^. Importin-α, also known as karyopherin-α, is an intracellular transporter protein that binds to GLUT2, functioning as an intracellular signal transducer^29,30^. Therefore, we hypothesized that importin-α mediates GLUT2-induced signaling and HNF-1α activation in PTs^31,32^. First, we identified importin isoforms that predominated in PTs. The importin isoforms α1, α5, and α7 were abundant compared with isoforms α3, α4, and α6, with the importin-α1 mRNA level being the highest (Fig. 4A). Under HG conditions, the expression pattern of each isoform of importin-α did not change (Supplementary Fig. 2). We used specific siRNA for importins-α1, -α5, and -α7 (Fig. 4B) and examined their roles in HNF-1α nuclear translocation. Adding a specific siRNA in the lower chamber HG medium for importin-α1, but not for -α5 or -α7, inhibited nuclear accumulation of HNF-1α (Fig. 4C). In addition, a specific siRNA for importin-α1 also blocked HG-induced SGLT2 expression upregulation (Fig. 4D). Finally, HNF-1α was immunoprecipitated with importin-α1 from lysates prepared from cells cultured with HG, but not in those cultured with NG medium (Fig. 4E). We immunoprecipitated GLUT2 with importin-α1 from lysates prepared from cells cultured with NG, but not from those cultured with HG medium (Fig. 4F). Taken together, ambient basolateral HG activates GLUT2/importin-α1/HNF-1α signaling, which resulted in SGLT2 expression upregulation in PT cells (Fig. 4G).

### Effects of Cana on SIRT1 expression in PT cells

**Figure 5:**
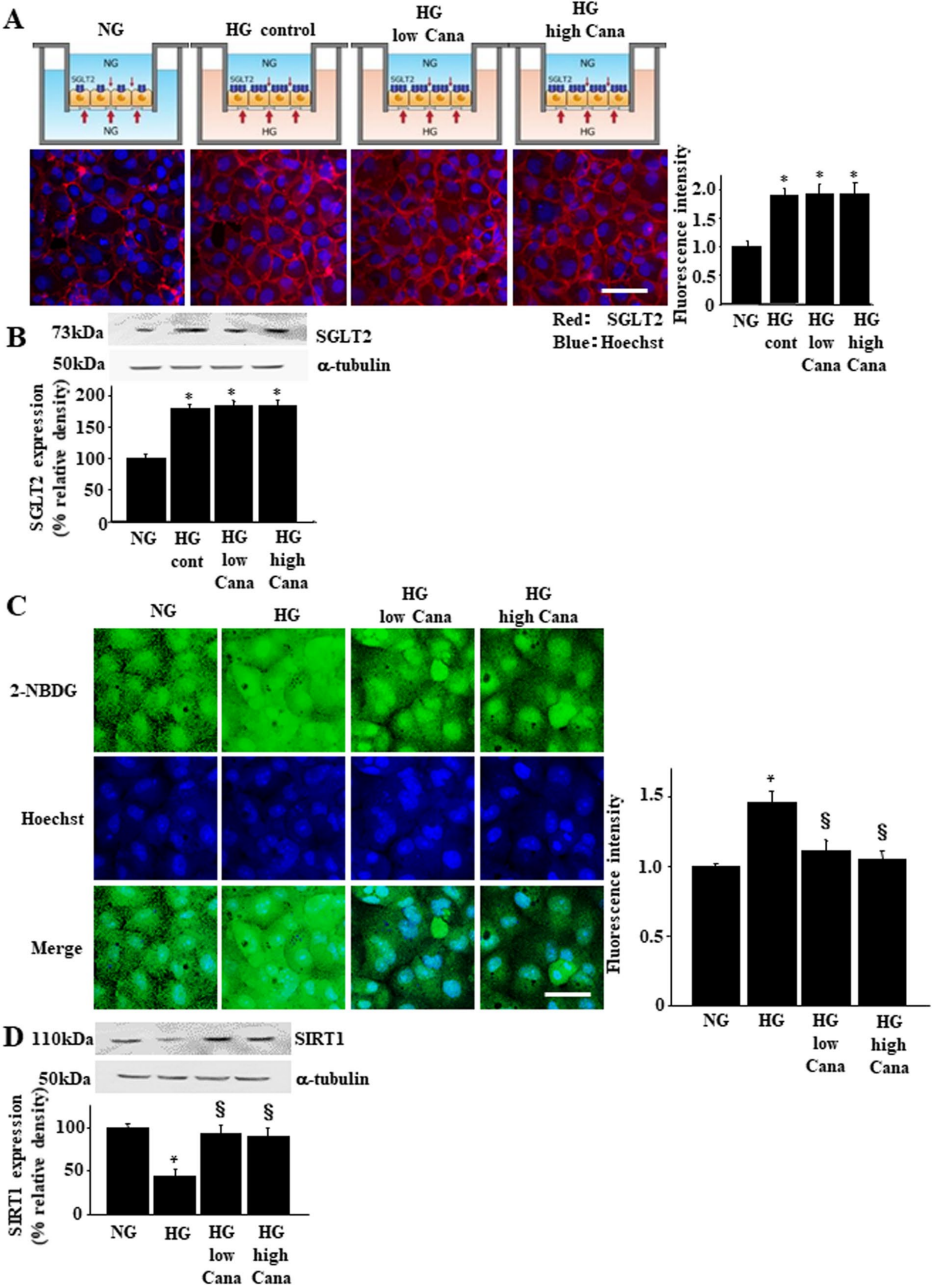
Effect of Cana on the expression of SIRT1 and its downstream signaling components. Confluent growth-arrested LLC-PK1 cell monolayers were stimulated with HG medium on the basolateral side for up to 24 h with or without pretreatment with Cana in the apical side. Immunofluorescence analysis (**A**) and immunoblotting (**B**) for SGLT2 expression in LLC-PK1 cells. The relative quantification of the SGLT2 immunofluorescence was measured and is indicated in the bar graphs. Scale bar, 50 µm. *P < 0.05 vs. NG group, *n* = 4 independent experiments. (**C**) The effect of SGLT2 inhibitors on cellular glucose entry in LLC-PK1 cells. LLC-PK1 cells were incubated in Dulbecco’s modified minimal essential medium containing 100 μM 2-NBDG for 15 min from the apical side of the cell. Cellular glucose entry was assessed as 2-NBDG entry into the cell, as described in the Methods section. Scale bar = 20 µm. *P < 0.05 vs. NG group and ^§^*P* < 0.05 vs. HG without Cana, *n* = 4 independent experiments. NG, normal glucose (5.5 mM); HG, high glucose (22.5 mM); HG + Cana, HG with Cana treatment. (**D**) Immunoblotting analysis of SIRT1 expression in LLC-PK1 cell monolayers stimulated with HG medium on the basolateral side for up to 24 h with or without pretreatment with low or high doses of Cana. Quantification of immunoblotting images was normalized to α-tubulin. *P < 0.05 vs. NG group and ^§^*P* < 0.05 vs. HG without Cana, *n* = 4 independent experiments.

Next, we studied effects of Cana on SGLT2 and SIRT1 expression using the two-chamber culture system. Because SGLT2 localizes to the apical side of PT cells, we added Cana to the upper chamber. Immunofluorescence and immunoblotting revealed that addition of HG to the basolateral chamber increased SGLT2 expression and 100 nM or 500 nM Cana did not inhibit this HG-induced increase in SGLT2 levels in LLC-PK1 cells (Fig. 5A,B). We monitored the entry of fluorescence-labeled glucose 2-NBDG in the upper chamber under the same experimental conditions^33^. Lower-chamber HG levels significantly increased glucose uptake from the upper chamber by LLC-PK1 cells, which was prevented by treatment with 100 or 500 nM Cana in the upper chamber (Fig. 5C). Lower-chamber HG levels significantly decreased SIRT1 expression. This process was inhibited by treatment with 100 or 500 nM Cana in the upper chamber (Fig. 5D).

### Correlation between SGLT2 and SIRT1 expression in human kidney

**Figure 6:**
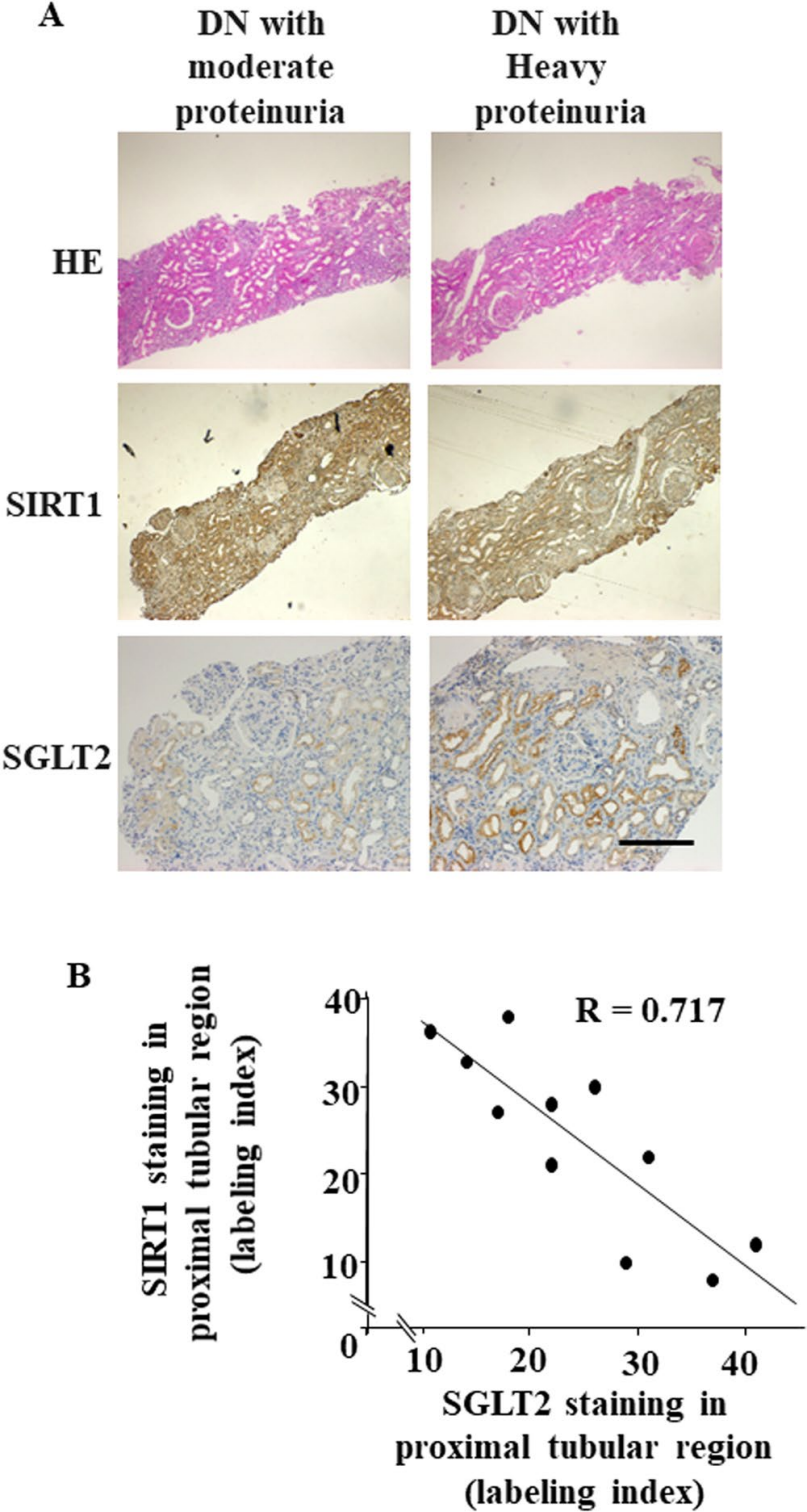
Immunohistochemical analysis of SIRT1 and SGLT2 expression in human renal biopsy specimens. (**A**) Representative photomicrographs of hematoxylin and eosin staining or immunostaining of SIRT1 and SGLT2 in renal needle-biopsy specimens of patients with diabetic nephropathy (DN) (DN-1 and -5, Table 1 for patient details). Scale bar, 50 nm. (**B**) The relationship between the intensity of immunostaining of SIRT1 and that of SGLT2 in the proximal tubular region in renal biopsy specimens from patients with DN, *n* = 11 subjects.

The negative correlation between SGLT2 and SIRT1 expression under diabetic conditions was further examined using kidney tissue samples from patients with diabetes. We completed immunostaining for SGLT2 and SIRT1 of renal specimens obtained from 11 patients with DN (Fig. 6A). The intensity of staining of these proteins was negatively correlated (Fig. 6B), suggesting that glucose entry via SGLT2 also downregulates kidney SIRT1 expression.

## Discussion

In DN, gene expression in renal epithelial cells, particularly in PT cells, is altered even during early stages^34^. In this study, we demonstrated increased SGLT2 expression and decreased SIRT1 expression in *db*/*db* mice. Cana treatment inhibited SIRT1 expression downregulation, indicating a close association between these molecular changes. We also used a two-chamber tissue culture system to show that SGLT2 expression upregulation is caused by HG conditions in the lower chamber, corresponding to HG levels in the basolateral side of PT cells. HG conditions did not affect SGLT2 expression upregulation in the upper chamber, which corresponds to the apical side of PT cells. HG conditions dissociated the GLUT2/importin-α1 complex, enhancing formation of importin-α1/HNF-1α complexes. Importin-α1 led to HNF-1α nuclear translocation, promoting SGLT2 transcription. Basolateral HG also downregulated SIRT1 expression and this was ameliorated by apical side Cana treatment. Under diabetic conditions, PTs increased SGLT2 expression, resulting in increased glucose entry into PTs from glomerular filtrates and thus decreased SIRT1 expression. This relationship was also observed in renal biopsy samples from patients with diabetes. These results suggest that glucose entry from the apical side by SGLT2 helps downregulate SIRT1 expression in patients with DN.

Extracellular glucose elicits several intracellular signals, including redox signaling and angiotensin II receptor-mediated signaling^22,23^. Our pharmacological experiments suggest that GLUT2-mediated signaling is a relevant pathway^20,21^. On the basolateral side of PT cells, GLUT2, a low-affinity and high-capacity transporter, transports glucose absorbed from the urinary lumen by SGLT2 to the interstitial space and peritubular capillaries in the kidney. GLUT2 is also a membrane sugar receptor^35^. For instance, in hepatoma cells, there is close correlation between GLUT2 levels and glucose-sensitive genes^36^. In a pancreatic β-cell line, glucose-induced insulin secretion was mediated by glucose influx through GLUT2^37^. This notion is supported by studies on GLUT2 null mice where the absence of GLUT2 impaired glucose-sensitive gene expression, including the pancreatic β-cell insulin gene^38–40^. GLUT2 appears to play a similar role in the glucose-sensitive SGLT2 gene expression in the kidney.

The molecular mechanism whereby GLUT2 directs intracellular signaling, resulting in the stimulation of glucose-sensitive gene transcription, was initially revealed in hepatocytes^29^. The large intracytoplasmic loop in the GLUT2 protein tethers the nuclear importer protein importin-α1, at the plasma membrane. Importin-α cooperates with importin-β and the cargo protein harboring the nuclear-localizing signal peptide as a nuclear import machinery, guiding target cargo molecules to shuttle through the nuclear pore^41^. We have identified several importin protein isoforms and this protein is a significant component of hepatocytes and pancreatic β-cells^41^. With extracellular glucose stimulation of mhAT3F hepatoma cells and pancreatic MIN-6 cells, massive amounts of importin-α1 accumulate in the cytoplasm^42^. These dynamic changes in the intracellular distribution were not detected in another liver cell line HepG2 or in a kidney cell line COS-7^42^. In addition, the signal-transduction role of each importin-α isoform appears to be cell- and tissue-specific^28^. In LLC-PK1 cells, importin-α1, -α5 and -α7 are dominantly expressed and only the α1 isoform is associated with HNF-1α nuclear translocation. Other reports have described dysregulation of the importin system under diabetic conditions^43^. In diabetes, we observed increased renal expression of all importin isoforms, although the pathological relevance of this increase remains unclear. We found that by binding to HNF-1α, importin-a1 plays a critical role in activating HNF-1α. Ambient glucose stimuli enhanced this binding during dissociation from the GLUT2 molecule (Fig. 4G). Thus, in diabetes, importin-α1 upregulation may exist as an adaptation to increases in extracellular glucose levels, contributing to enhanced HNF-1α transport into the nucleus and resulting in SGLT2 expression upregulation.

The HNF-1α transcription factor acts as a chaperone protein for importin 1α. HNF-1α is required for glucose metabolism in the liver, pancreatic islets, kidneys, and intestines^44^. HNF-1α-deficient mice and patients suffering from maturity-onset type 3 diabetes develop type 2 diabetes and renal Fanconi syndrome characterized by increases in urinary glucose levels^45^. As demonstrated in this study, HNF-1α directly upregulates SGLT2 transcription, thereby contributing to renal glucose reabsorption. Although three regions within the HNF-1α gene encode NLS^32^ and HNF-1α shuttles between the nucleus and cytoplasm^31^, molecules that interact with NLS as well as the mechanisms for HNF-1α nuclear translocation remain unknown. This study is the first to demonstrate that importin-α1 transports HNF-1α to the nucleus, resulting in SGLT2 transcription upregulation (Fig. 4G).

Previous studies indicated that ambient HG decreases SIRT1 activity and expression in various cell types^46–48^, although detailed mechanisms are unknown. Increased oxidative stress, provoked by glucose entering the cell, plays a key role in this regulation. Increases in oxidative stress reduce cellular concentration of NAD^+^, leading to decreased SIRT1 activity. This, in turn, inactivates the forkhead box O1 (FoxO1) transcription factor, which then downregulates SIRT1 transcription^49^. Redox-sensitive transcription co-repressor CtBP may also inhibit SIRT1 transcription^50^. ROS degrade SIRT1 proteins through a proteasome-dependent mechanism^51^. In the present study, entry of glucose from the apical side was important for SIRT1 downregulation because upper chamber treatment with an SGLT2 inhibitor completely mitigated HG-induced downregulation of SIRT1. In other words, glucose entry because of HG in the lower chamber had little effect on SIRT1 expression, indicating that glucose entry from the basolateral side, through GLUT2, was marginal. Given that GLUT2 facilitates glucose transport, glucose entry occurs by simple diffusion and depends on the glucose concentration gradient inside and outside the cell. In contrast, SGLT2 is driven by electrochemical sodium ions gradients, and as long as sodium-potassium dependent ATPase pumps sodium out, glucose entry ensues, irrespective of the glucose gradient. Therefore, in renal PT cells, blocking SGLT2 prevents excessive glucose entry and glucose toxicity.

In diabetic conditions, clinical significance of restoring proximal tubular SIRT1 by SGLT2 inhibition merits comment. In addition to its plasma glucose-lowering effects, there are several beneficial renal effects of SGLT2 inhibition. SGLT2 inhibitors ameliorate DN by reducing oxidative stress in the renal tubules of *db*/*db* mice. We previously reported renal protective effects of SIRT1 through upregulation of the anti-oxidative stress molecule catalase in proximal tubular cells^51^. Several other mechanisms are involved in SIRT1-related anti-oxidative stress effects in the kidney^52^. SGLT2 inhibitors exhibit anti-inflammatory effects in mice with diabetes. By deacetylating the p65 subunit, SIRT1 also inhibits NFκB activity and kidney tissue inflammatory responses^53^. Moreover, SIRT1 protects against diabetic albuminuria by epigenetic podocyte claudin-1 downregulation^4^. SGLT2 inhibition also reduces albuminuria in mice with diabetes^6^. SIRT1 expression may explain several favorable renal effects demonstrated by SGLT2 inhibition.

As for the validity of a SGLT2 antibody used in this study, we have not perfiormed studies using SGLT2 knockout mice. However, we confirmed that there was no extra-renal SGLT2 expression (Supplementary Fig. 19A), supporting the specificity of the antibody. In addition, we assured antibody specificity using siRNA-mediated gene silencing (Supplementary Fig. 19B,C). There are several studies using this commercial antibody^54–56^.

Finally, SGLT2 inhibition reduces mortality in high-risk patients with diabetes^57^. SIRT1 is known as a “longevity gene,” and its introduction elongates the lifespan of yeast, hook worms, drosophila, rodents, and mammals^58^. SGLT2 inhibition effects on longevity may be related to SIRT1 activation, which favors tissue resistance to various stresses in renal and other SGLT2-expressing tissues.

In conclusion, SGLT2 expression was increased in the kidneys of *db*/*db* mice and humans with diabetes, whereas SIRT1 expression was decreased. SGLT2 expression upregulation results from basolateral glucose stimulation activating the GLUT2/importin-α1/HNF-1α signaling pathway. Cana-related inhibition of SGLT2 restored SIRT1 expression by preventing intracellular glucose entry from the apical side into the proximal tubular cells. Thus, Cana may be a beneficial treatment strategy against diabetic kidney disease.

## Electronic supplementary material

Supplementary Text and Figures
