# Supplementary material for: High Basolateral Glucose Increases Sodium-Glucose Cotransporter 2 and Reduces Sirtuin-1 in Renal Tubules through Glucose Transporter-2 Detection
Source: Sci Rep. 2018 May 1;8:6791. doi: 10.1038/s41598-018-25054-y (PMC5931531; doi:10.1038/s41598-018-25054-y)
Supplement: Supplementary file 1 — Supplementary Text and Figures [file 41598_2018_25054_MOESM1_ESM.pdf]

High Basolateral Glucose Increases Sodium-Glucose CoTransporter 2 and Reduces Sirtuin1 in Renal Tubules through Glucose Transporter-2 Detection

Supplementary materials

Hiroyuki Umino[1, #], Kazuhiro Hasegawa[1, #], Hitoshi Minakuchi[1], Hirokazu Muraoka[1], Takahisa Kawaguchi[1], Takeshi Kanda[1], Hirobumi Tokuyama[1], Shu Wakino[1] and Hiroshi Itoh[1]

# These authors contributed equally to this work.

[1] Department of Internal Medicine, School of Medicine, Keio University, Tokyo, 160-8584, Japan

Corresponding Author:

Shu Wakino, M.D.

Department of Internal Medicine, School of Medicine, Keio University

35 Shinanomachi, Shinjuku-ku, Tokyo 160-8582, Japan

Tel: +81-3-5363-3796

Fax: +81-3-3359-2745

E-mail: shuwakino@z8.keio.jp

SUPPLEMENTARY METHODS

Histopathological analysis

Immunohistochemistry was performed as previously described.[1] Briefly, paraffin sections (4 μm) were fixed in 3% formaldehyde and stained using primary SGLT2 (1:50; Abcam Inc., Cambridge, MA, USA) and SIRT1 antibodies (1:100; Millipore, Bedford, MA, USA). The sections were then incubated with biotin-labelled goat anti-rabbit IgG (1:200; Vector Laboratories, Burlingame, CA, USA) or anti-mouse IgG (1:200; Vector Laboratories) antibodies and then treated with the Vectastain Elite ABC Kit (Vector Laboratories). Sections were examined using an Olympus BX53 biological microscope (Oympus Corporation, Tokyo, Japan). In a kidney histological quantitation by a blinded observer, a minimum of 15 non-overlapping images of cortex per kidney were observed under ×200 magnification. Quantitative computer-assisted image analysis was performed by a blinded observer by using the Image-Pro Plus software (Media Cybernetics, Bethesda, MD).

Immunofluorescence against SGLT2 in LLC-PK1 cells

LLC-PK1 cells were seeded in chamber slides and treated under the indicated conditions. Cells were washed with ice-cold phosphate-buffered saline (PBS) and fixed with 4% paraformaldehyde in PBS, pH 7.4, at 22°C for 20 min, then permeabilised with

0.1% Triton X-100 in PBS for 5 min. Cells were treated with 5% goat serum at 22°C for 1 h to minimise nonspecific antibody binding and incubated with the anti-SGLT2 antibody (1:50; Abcam Inc.) in 2.5% goat serum overnight at 4°C. After washing, cells were incubated with a fluorescently-tagged secondary antibody for 1 h at 22°C in the dark. Nuclei were stained with 10 nM DAPI and samples were mounted using ProLong Gold antifade mounting medium (Invitrogen, Eugene, OR, USA). Images were acquired at 22°C using an Olympus IX-81 microscope (Olympus Corporation, Tokyo, Japan) equipped with an Olympus SpectraMaster II monochromator light source and UltraPix camera (Perkin Elmer Corporation, Boston, MA, USA) equipped with a PlanApo 60×/1.40 oil-immersion objective. Images were analysed using UltraView software (PerkinElmer). LLC-PK1 cells (passages 8–30) were used for cell culture in the present study.

2-NBDG incorporation by LLC-PK1 cells.

The glucose biosimilar molecule, 2-deoxy-2-[(7-nitro-2,1,3-benzoxadiazol-4-yl) amino]-D-glucose (2-NBDG), was used to detect glucose incorporation into LLC-PK1 cells. Cells were seeded into six-well culture plates and cultured in complete medium that was replaced every other day. 2-NBDG was added on day 5. Fresh medium

containing 2-NBDG (500 μM) was added to the wells, and 3 h later, cells were washed 3× with 500 μL ice-cold PBS and then placed on microscope slides for analysis. Confocal fluorescence microscopy was performed using a Zeiss Axiovert 200M inverted microscope (Carl Zeiss, Ltd., Welwyn Garden City, UK).

Immunoprecipitation analysis

Immunoprecipitation was performed as previously described according to the manufacturer's protocol (Roche Applied Science, Indianapolis, IN, USA). Briefly, we incubated 500 μg of cellular lysates total protein with protein A-agarose beads (Roche Applied Science) for 1 h to clear the samples. After centrifuging the samples, the supernatant was collected and incubated with a rabbit anti-importin-α1 antibody (5 μg, Cell Signaling Technologies) overnight at 4°C with gentle agitation. Lysates were incubated with protein A-agarose beads for 3 h at 4°C, and after washing, the beads were boiled to release the bound proteins, which were separated using SDS-PAGE and immunoblotted.

Immunoblotting of mouse kidney tissues and LLC-PK1 cells

Immunoblotting was performed using antibodies against SIRT1 (1:1,000; Millipore),

SGLT2 (1:100; Abcam Inc.), GLUT2 (1:1000; Millipore), HNF-1α (1:500; Santa Cruz Biotechnology), importin-α1 (1:1000; MBL, Nagoya, Japan), importin-α5 (1:1000; Proteintech) and importin-α7 (1:500; Abcam Inc.). α-tubulin (1:1000; Abcam Inc.) and poly ADP ribose polymerase (1:1000; Abcam Inc.) were used as loading controls. Band intensities were quantified using Scion Image Software (Scion Corp., Frederick, MD, USA).

Quantitative Real-time PCR

Approximately 600 ng of total RNA was used for reverse transcriptase (RT) reactions, which were performed using the Superscript pre-amplification system (Invitrogen). The RT product was used for real-time quantitative PCR amplification of mRNAs encoding importin-α isoforms using a LightCycler System (Roche) in the presence of LightCycler FastStart DNA SYBR Green I master mix (Roche), using importin-α isoform-specific primers. The primer sequences for forward and reverse primers respectively were as follows: importin-α1, 5′-GTGTCCTTCTTGGGCAGAAC-3′ and 5′-ATGGCACCTCCATCTACCAC-3′; importin-α3, 5′-TGTGAGCAAGCAGTGTGGGCA-3′ and 5′-TGGTGGTGGGTCTTTGTGGCG-3′; importin-α4, 5′-ATCCCCCGCCGCCTATGGAG-3’ and

5′-CTGGGTCTGCTCGTCGGTGC-3′; importin-α5, 5′-AGTAGTGGCCAGGTTTGTGG-3′ and 5′-TGAATCACAATTCGGGTCTG-3′; importin-α6, 5′-GTGATTGAAACTGGGGCTGT-3′ and 5′-CTGCATTCTGCATTGTCACC-3′, importin-α7, 5′-ACTCCAAGAGTGGTGGATCG-3′ and 5′-GGTCTGCTGAGAGGTTCCAG-3′; glyceraldehyde 3-phosphate dehydrogenase (GAPDH), 5′-AGGGCTGCTTTTAACTCTGGT-3′ and 5′-CCCCACTTGATTTTGGAGGGA-3′.

Importin siRNA transfection

Small interfering RNAs (siRNAs) corresponding to importins-α1, -α5 and -α7 were designed using BLOCK-iT RNAi Designer (Invitrogen). The siRNA sequences targeting importins-α1, -α5 and -α7 were CCAAGCUACUCAAGCUGCCAGGAAA, CCGGAAUGCAGUAUGGGCUUUGUCU and CAGCCCUACCUUGCCUUCUCCACUU, respectively. LLC-PK1 cells were seeded one day before transfection with the siRNAs. On day 0, transfection of *importin-α1-*, *-α5-* and *-α7*-specific siRNAs, cells were treated with Lipofectamine RNAiMAX (Invitrogen) and transfected with a nonspecific siRNA (negative control) following the supplier's protocol. After 2 days, second transfections were performed, and 2 days later

the cells were harvested and cell lysates prepared. Transfection efficiencies of the siRNAs were determined using western blotting with monoclonal antibodies against importins-α1, -α5 and -α7.

Metformin experiments

Metformin was purchased from Sigma-Aldrich (St. Louis, MO, USA). Eight-week-old, male db/m+ (C57BLKS/J lar-m+/ Leprdb) and db/db (C57BLKS/J lar-Leprdb/Leprdb) mice were used in the experiments. After acclimatization to their environment for 7 days, the mice were randomized into four different groups, with 4 animals/group (n = 8). Two groups were not treated with metformin: *db/m* without metformin (*db/m c*ontrol) and *db/db* without metformin (*db/db* control). The other two groups were administered metformin: *db/db* with low-dose metformin (*db/db* low Met) and *db/db* with high-dose metformin (*db/db* high Met). The metformin concentrations in the diets of the mice were high (300 mg/kg body weight) or low (150 mg/kg body weight), as per the values suggested by the literature on metformin in mice (20–23), which were in turn calculated from the average food consumption (8 g/day) and the average body weight (40 g or 0.04 kg) of the diet-induced mice. The final calculation came to be equivalent to 1.5 g and 0.75 g metformin/kg diet, respectively. Therefore, the average daily dose of the drug

(calculated from the food intake and body weight) was as follows: 0.15% metformin w/w food admixture = 300 mg/kg and 0.075% metformin w/w food admixture = 150 mg/kg.

Blocking peptides

The blocking peptides were obtained from the supplier. In western blotting, the sample was pre-incubated with the blocking peptides at a final concentration of 0.5 mg/mL for 4 h at the room temperature and then used in the immunoblotting assay, as described previously (2). For immunostaining process, we applied the blocking peptides at a final concentration of 0.5 mg/mL for 4 h at the room temperature prior to use in the previously described immunostaining procedure.

Extrarenal SGLT2 protein expression in wild-type mice

Adult male C57BL/6 mice (3 month old) were used for testing SGLT2 protein expression in WT mice. Various organs were rapidly harvested and immediately frozen in liquid nitrogen for analyses at another time. The method of Western blotting has been described in the main text. An antibody against β-actin (1:500; Abcam Inc.) was used as a loading control.

SGLT2 siRNA transfection

siRNA was designed to target sites specific for SGLT2 mRNA based on the porcine SGLT2 sequence (accession no: NC_010445.4). SGLT2 siRNA (sense, 5′-CUU UAU UUU UUA UUU UUG UdTdT-3′ and antisense, 5′-ACA AAA AUA AAA AAU AAA GdGdT-3′) was used. Allstars Negative Control siRNA was used as a negative control (Qiagen, Mississauga, ON, Canada). This control siRNA had no homology to any known mammalian gene. siRNA transfection protocol was the same as that described for importin siRNA transfection.

SGLT2 mRNA detection

Primers were designed and synthesized with reference to previous studies using LLCPK-1 cells (3, 4) as follows: 5′CCAATAGAGGCACAGTTGGTGG (forward) and 5′GCGTAAATGTTCCAGCCCAGG (reverse) for SGLT2. Quantitative real-time PCR was performed as described in the main text.

SUPPLEMENTARY REFERENCE

1. Roxburgh, S. A. *et al.* Allelic depletion of grem1 attenuates diabetic kidney disease.

Diabetes. **58**, 1641-1650 (2009).

2. Sabolic I. *et al.* Expression of Na+-D-glucose cotransporter SGLT2 in rodents is kidney-specific and exhibits sex and species differences. *Am J Physiol Cell Physiol*. **302**, C1174-88 (2012).

3. Maldonado-Cervantes, M. I. *et al.* Autocrine modulation of glucose transporter SGLT2 by IL-6 and TNF-α in LLC-PK(1) cells. *J. Physiol. Biochem*. **68**, 411-20 (2012).

4. Zapata-Morales, J. R. *et al.* Hypoxia-inducible factor-1α (HIF-1α) protein diminishes sodium glucose transport 1 (SGLT1) and SGLT2 protein expression in renal epithelial tubular cells (LLC-PK1) under hypoxia. *J. Biol. Chem.* **289**,346-57 (2014).

SUPPLEMENTARY FIGURE LEGENDS

Supplementary Figure S1. Effect of Cana on GLUT2 expression in *db/db* mice.

GLUT2 immunostaining in kidney sections is shown. Scale bars 500 μm (low power field, LPF, ×100). The relative quantification of GLUT2 expression was measured and is indicated in the bar graphs. n = 8 mice/group.

Supplementary Figure S2. The expression of human importin isoforms under HG conditions.

Quantitative real-time PCR analysis of the relative abundance of mRNAs encoding

human importins in LLC-PK1 cells under HG conditions. Real-time PCR data were normalised to those of the *GAPDH* mRNA. The relative fold-differences were calculated using the mean value (*n* = 6 independent experiments) of importin-α6.

Supplementary Figure S3–6. High magnification of GLUT2 immunostaining.

GLUT2 immunostaining of the kidney sections. Scale bars = 100 μm (high power field [HPF], ×400). Supplementary Fig S3 shows higher magnification of db/m in Supplementary Fig S1, Fig S4 correspond with db/db, Fig S5 exhibits db/db + low Cana, and Fig S6 demonstrates db/db + high Cana. These HPF figures demonstrate that GLUT2 is expressed in the basolateral membrane. n = 8 mice/group.

Supplementary Figure S7–10. Low and high magnification of Sglt2 immunostaining. The kidneys were immunostained for anti-Sglt2 antibody as described under the Experimental Procedures. Original magnification was ×100 (*LPF*) or ×400 (*HPF*). Scale bars = 500 μm (low power field [LPF]) and 100 μm (high power field [HPF]). The kidney immunostaining demonstrates that Sglt2 is localized in the apical membrane. n = 8 mice/group.

Supplementary Figure S11. *A*) Immunohistochemical labelling of Sglt2 antibodies on

mouse renal sections. Sglt2 antibody was tested against renal sections from db/m mice and pre-incubated with the blocking peptide. Original magnification was ×100 (*LPF*) or ×400 (*HPF*). Scale bars = 500 μm (low power field [LPF]) and 100 μm (high power field [HPF]). *B)* Western blotting using a sglt2 antibody. A sglt2 antibody was tested against the kidney lysate from *db/m* mice with and without pre-adsorption with its blocking peptide (*BP)*. The lower lane represents α-tubulin antibody, which was used as a loading control.

Supplementary Figure S12. Effect of metformin on parameters of glucose metabolism. *A*) Schedule for metformin (Met) treatment and experimental groups. The effects of Met on body weight **(***B*), food intake **(***C*), fasting plasma glucose concentration (*D*), and glycated Hb levels (*E*) in *db/db* mice. HbA1c, haemoglobin A1c. Data represent the mean ± SEM. * P < 0.05 vs. *db/m*, § P < 0.05 vs. *db/db*. n = 8 mice/group.

Supplementary Figure S13–14. Effect of Met on SGLT2 and SIRT1 expression in *db/db* mice. S13; *A*) Immunohistochemical analysis using a SGLT2-specific antibody. Representative kidney sections are shown for each group of mice. Quantitation of the

relative density is shown in the bar graph in the right panel. Scale bar = 100 µm. n = 8 mice/group. *B*) The upper panel shows the representative immunoblotting analysis of the SGLT2 expression. The bar graph in the lower panel indicates the quantification of the SGLT2 levels. The protein expression was normalised to that of α-tubulin. The relative protein levels are shown as the fold-change with respect to the *db/m* (control) group. * $P < 0.05$ vs. *db/m* control, §$P < 0.05$ vs. *db/db* control, $n = 3$ independent experiments. S14; A) Immunohistochemical analysis using a SIRT1-specific antibody. Representative kidney sections are shown for each group of mice. Quantitation of the relative density is shown in the bar graph. Scale bar = 50 µm. * $P < 0.05$ vs. *db/m* control, § $P < 0.05$ vs. *db/db* control, $n = 8$ mice/group. B) The upper panel shows the representative immunoblotting analysis of the SIRT1 expression. The bar graph in the lower panel indicates the quantification of the SIRT1 levels. The protein expression was normalised to that of α-tubulin. The relative protein levels are shown as the fold-change to the *db/m* (control) group. * $P < 0.05$ *db/m* control, § $P < 0.05$ vs. *db/db* control, $n = 3$ independent experiments.

Supplementary Figure S15–16. Effect of the GLUT2 inhibitor phloretin on the expression of SIRT1 and its downstream signalling components. S15; *A*) Confluent

growth-arrested LLC-PK1 cell monolayers were stimulated with HG medium on the basolateral side for up to 24 h with or without pretreatment with phloretin in the apical side. Immunofluorescence analysis (*A*) and immunoblotting (*B*) for the SGLT2 expression in LLC-PK1 cells. The relative quantification of the SGLT2 immunofluorescence was measured and is indicated in the bar graphs. Scale bar = 50 µm. *$P < 0.05$ vs. NG group, §$P < 0.05$ vs. HG without phloretin, $n = 3$ independent experiments. **S16; A**) The effect of phloretin on cellular glucose entry in LLC-PK1 cells. The LLC-PK1 cells were incubated in Dulbecco's modified minimal essential medium containing 100 µM 2-NBDG for 15 min from the apical side of the cell. Cellular glucose entry was assessed as 2-NBDG entry into the cell, as described in the Methods section. Scale bar = 20 µm. NG, normal glucose (5.5 mM); HG, high glucose (22.5 mM); HG + phloretin, HG with phloretin treatment. B) Immunoblotting analysis of the SIRT1 expression in LLC-PK1 cell monolayers stimulated with HG medium on the basolateral side for up to 24 h with or without pretreatment with low or high doses of phloretin. The quantification of immunoblotting images was normalised to that of α-tubulin. * $P < 0.05$ vs. NG group, § $P < 0.05$ vs. HG without phloretin, $n = 3$ independent experiments.

Supplementary Figure S17. Effect of high glucose concentration or osmotic control substance, mannitol on the Sglt2 expression. The results for cells grown in normal glucose (NG), in high glucose (HG), and exposed to mannitol (Mann). *P < 0.05 vs. NG, **§**P < 0.01 vs. HG. Data of three independent experiments are expressed.

Supplementary Figure S18. Localization of protein markers for apical and basal polarity. LLCPK-1 cells show E-cadherin localized to the basal membrane and NHE3 localized to the apical membrane. *Top plane*, the slice farthest from the filter support used to grow the cells. *Bottom plane*, the slice near the filter support (attached surface). Data of three independent experiments are expressed.

Supplementary Figure S19. The specificity of a SGLT2 antibody. A) Absence of SGLT2 protein expression in extrarenal organs of the mouse. Western blot analysis was performed in protein lysates of various organs from male WT mice. Our SGLT2 antibody recognized a protein band corresponding to the SGLT2 protein in the kidneys of WT mice, whereas the studied extrarenal organs showed no band intensities. Each lane was loaded with 10 μg of whole organ lysate. β-actin was used to confirm equal loading, although its expression varied slightly between organs. Results are

representative of three independent experiments. **B, C)** Antibody specificity was confirmed using siRNA-mediated gene silencing. SGLT2 siRNA and control siRNA were transfected into LLC-PK1 cells, and the SGLT2 protein expression was assessed using western blotting. **B)** Quantitative real-time PCR analysis of the relative abundance of SGLT2 mRNAs in LLC-PK1 cells. Real-time PCR data were normalized to those of the *GAPDH* mRNA. Results are representative of four independent experiments. **C)** LLC-PK1 cells were transiently transfected with siRNAs targeting GLUT2 or a non-targeting control siRNA. Whole cell lysates were prepared 48 h post-transfection and analyzed using immunoblotting. Results are representative of four independent experiments. The bar graph represents the band intensity of each group (* $P < 0.05$ vs. NG group, n = 4).

Supplementary Figure S20. Effects of Glut1-siRNA (100 nmol/L) or non-targeting control siRNA (100 nmol/L) on SGLT2 expression. Results are representative of four independent experiments. The bar graph in the right panel indicates quantification of SGLT2 levels. The protein expression was normalized to that of α-tubulin. Relative protein levels are shown as the fold-change to the NG group. * $P < 0.05$ vs. NG group, n = 4 independent experiments.

Supplementary Figure S21. The reduced nuclear HNF1α in GLUT2 siRNA.

Subcellular fractionation and western blotting demonstrate HG-induced redistribution from the cytoplasm to the nucleus of HNF-1α. HG, high D-glucose (22.5 mM) condition; NG, normal D-glucose (5.5 mM) condition; N, nuclear fraction; C, cytosolic fraction. HNF-1α-protein levels were determined by a densitometry and are indicated for each fraction. The percent of HNF-1α-in the nucleus relative to the total HNF-1α-was calculated using values determined by a densitometry. LLC-PK1 cells were transfected with the indicated siRNA duplexes, and 48 h later, cells were treated with media containing high glucose levels. * $P < 0.05$ vs. NG group, § $P < 0.05$ vs. HG group, and ¶ $P < 0.05$ vs. HG with GLUT2 siRNA, n = 4 independent experiments.

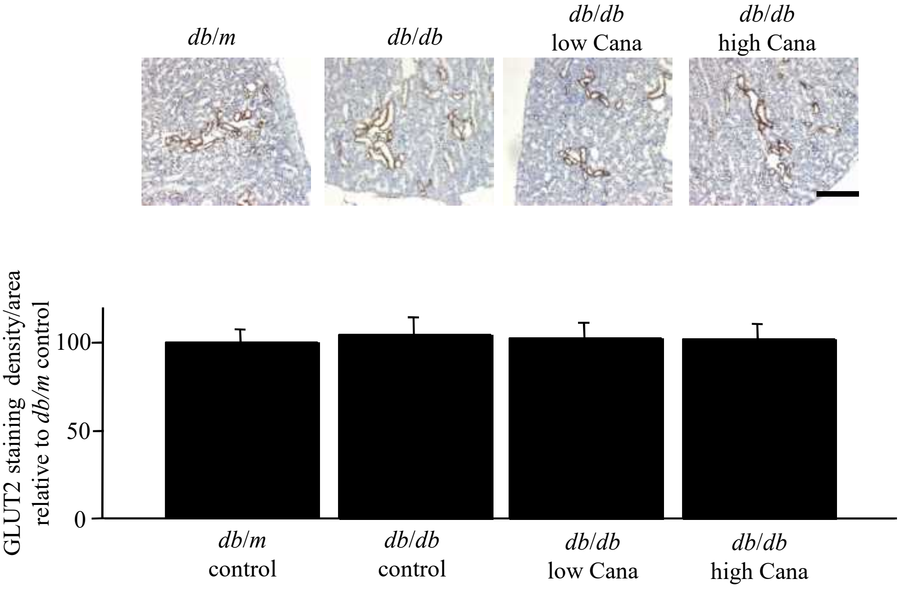

Supplementary figure 1

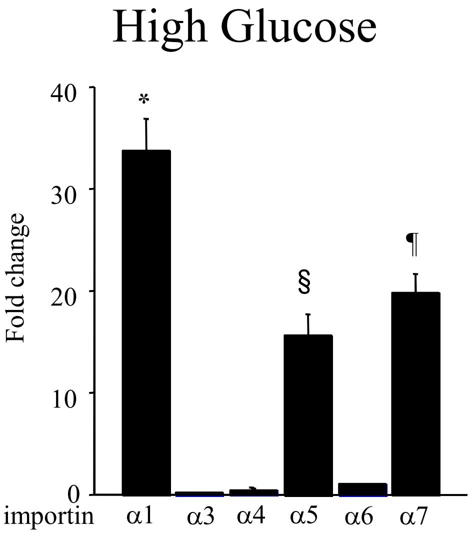

Supplementary figure 2

GLUT2 staining (high magnification)

db/m

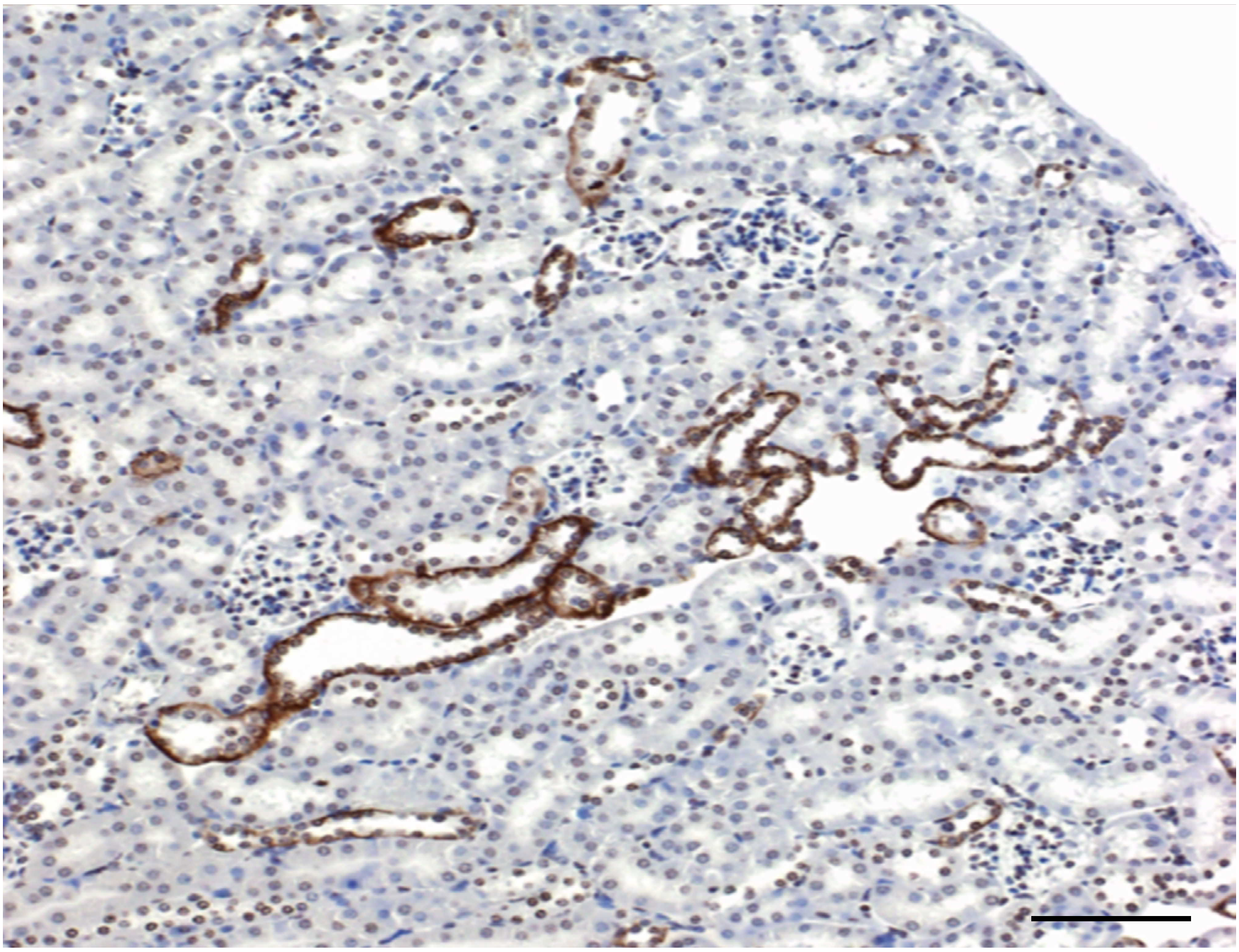

Supplementary figure 3

GLUT2 staining (high magnification)

db/db

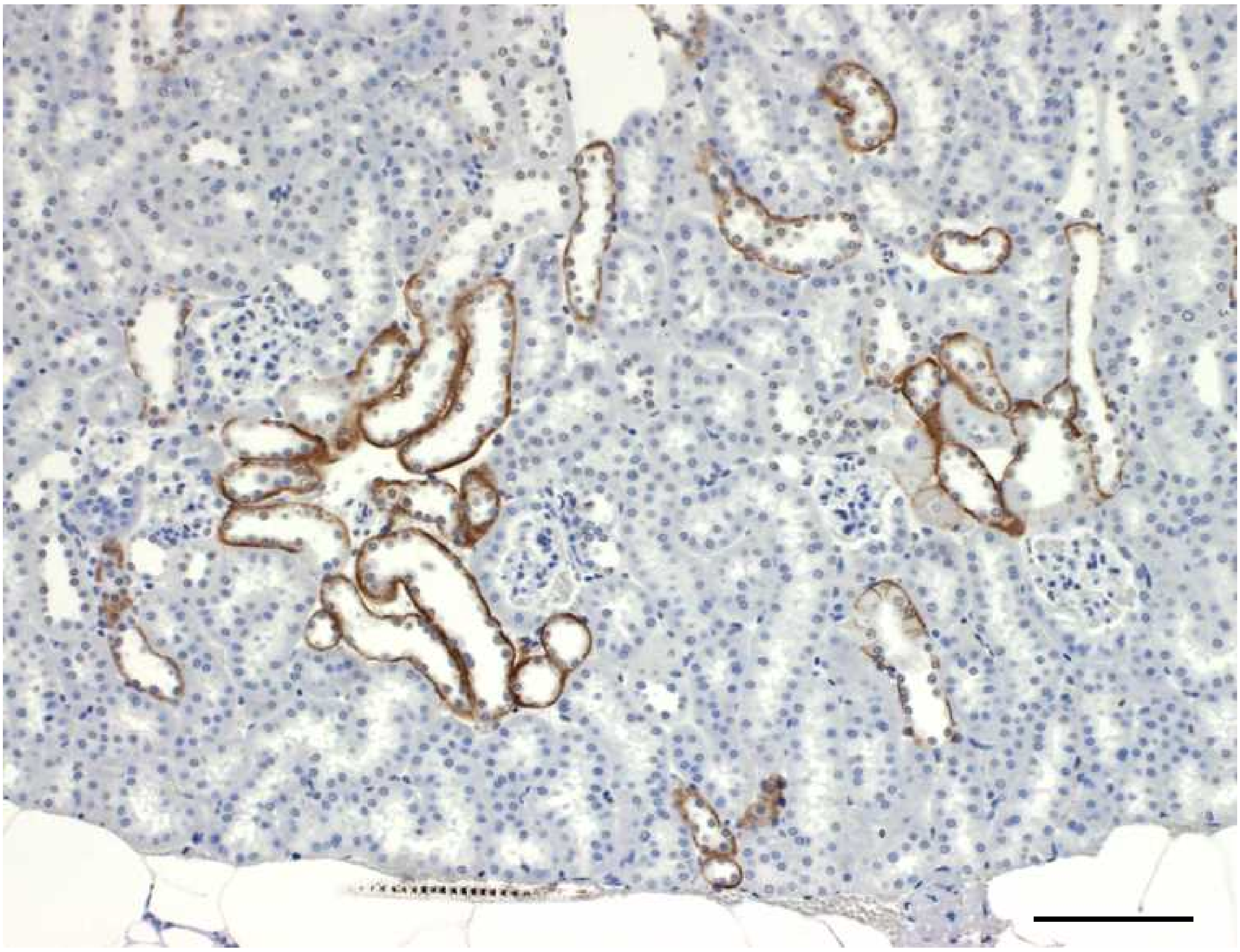

GLUT2 staining (high magnification)

db/db low Cana

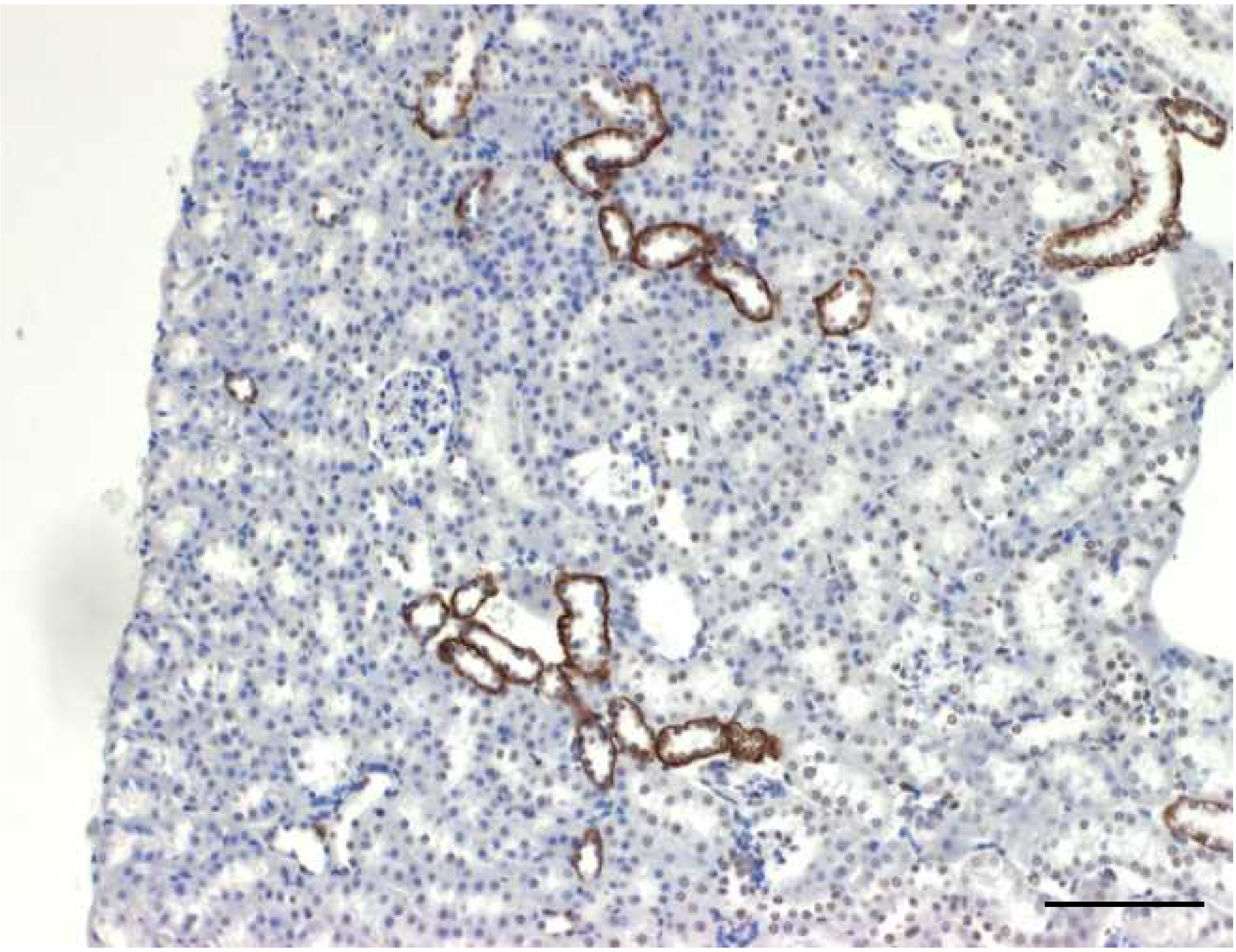

Supplementary figure 5

GLUT2 staining (high magnification)

db/*db* high Cana

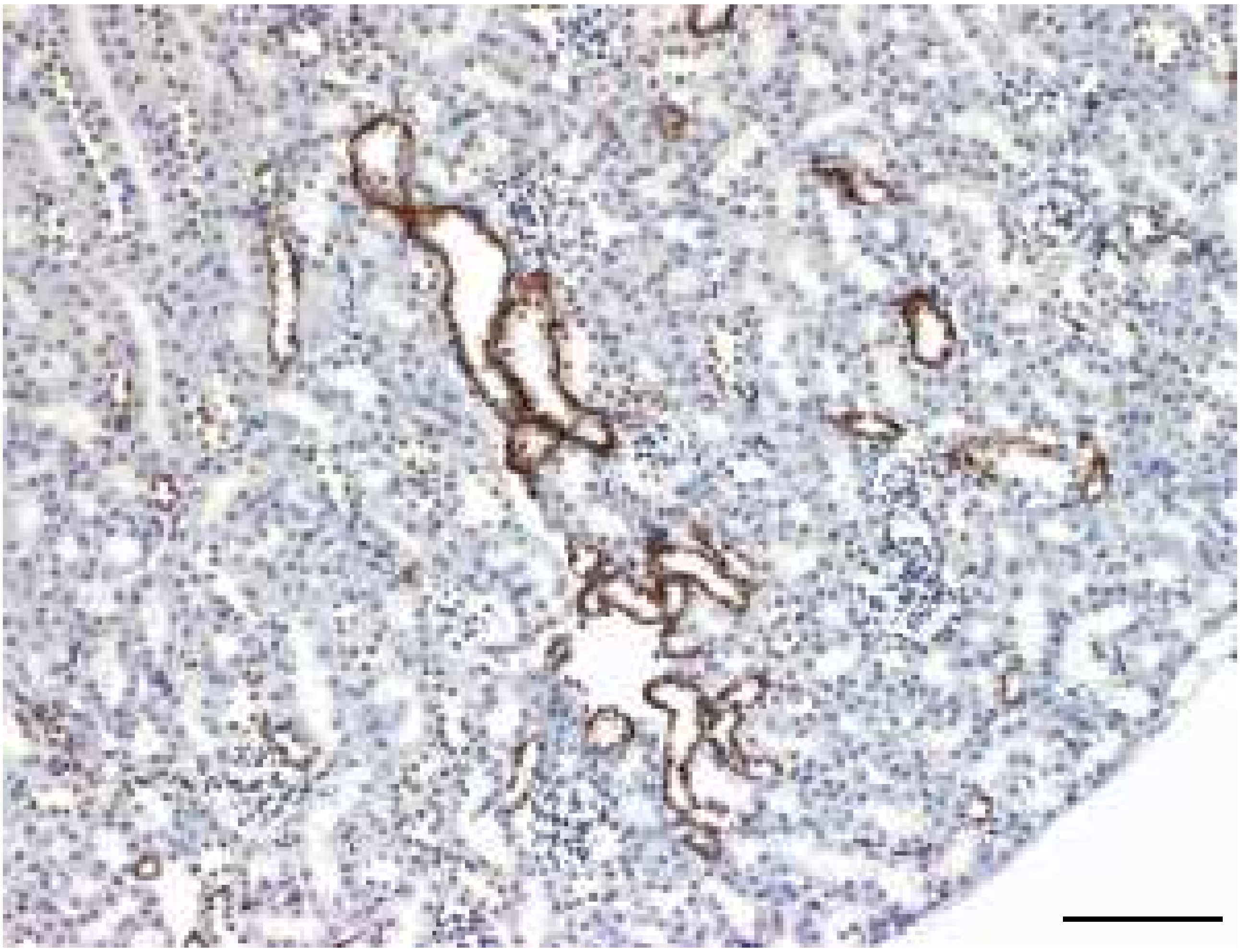

Supplementary figure 6

Sglt2 staining (low and high magnification)

db/m

LPF

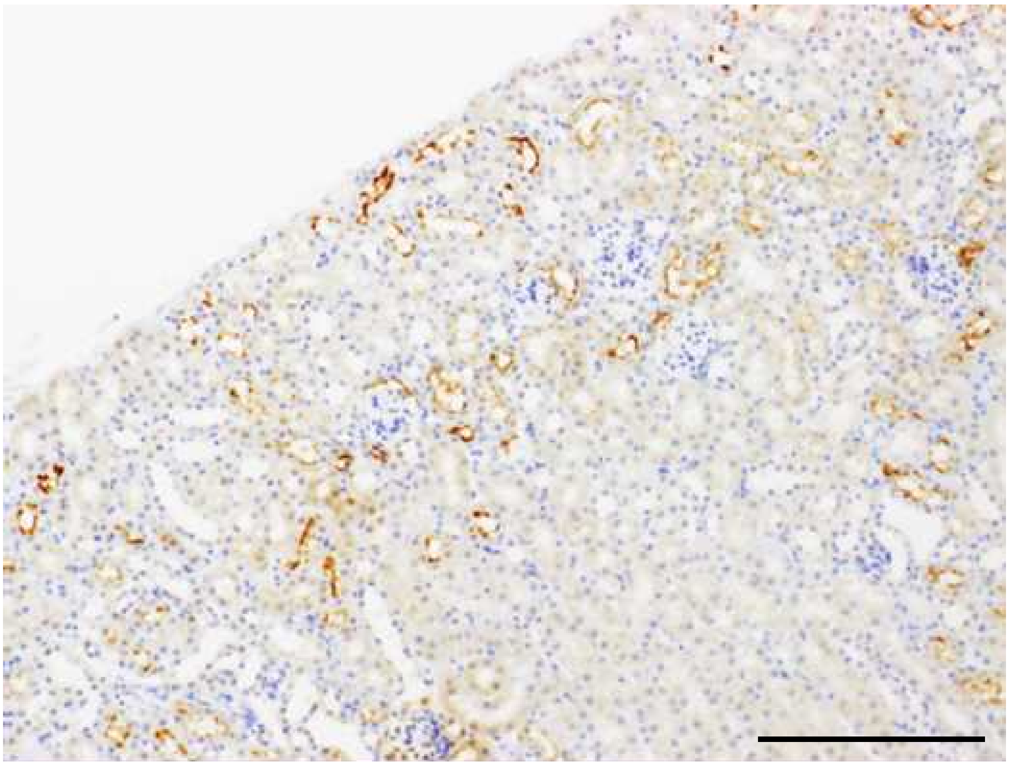

HPF

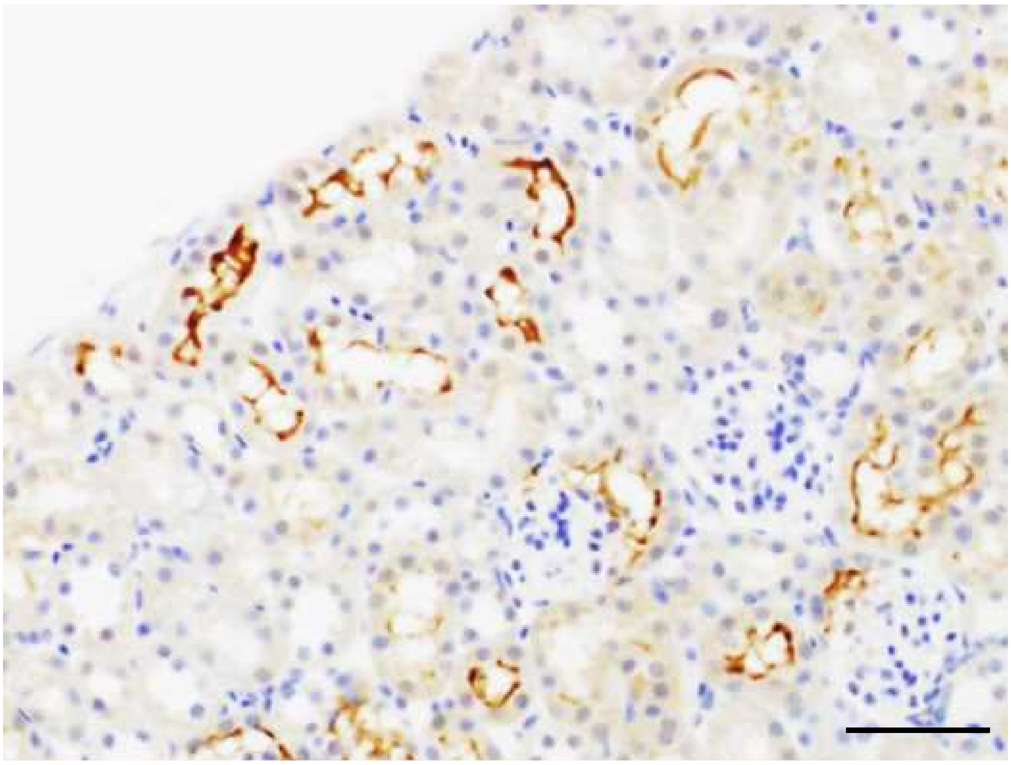

Supplementary figure 7

Sglt2 staining (low and high magnification)

db/db

LPF

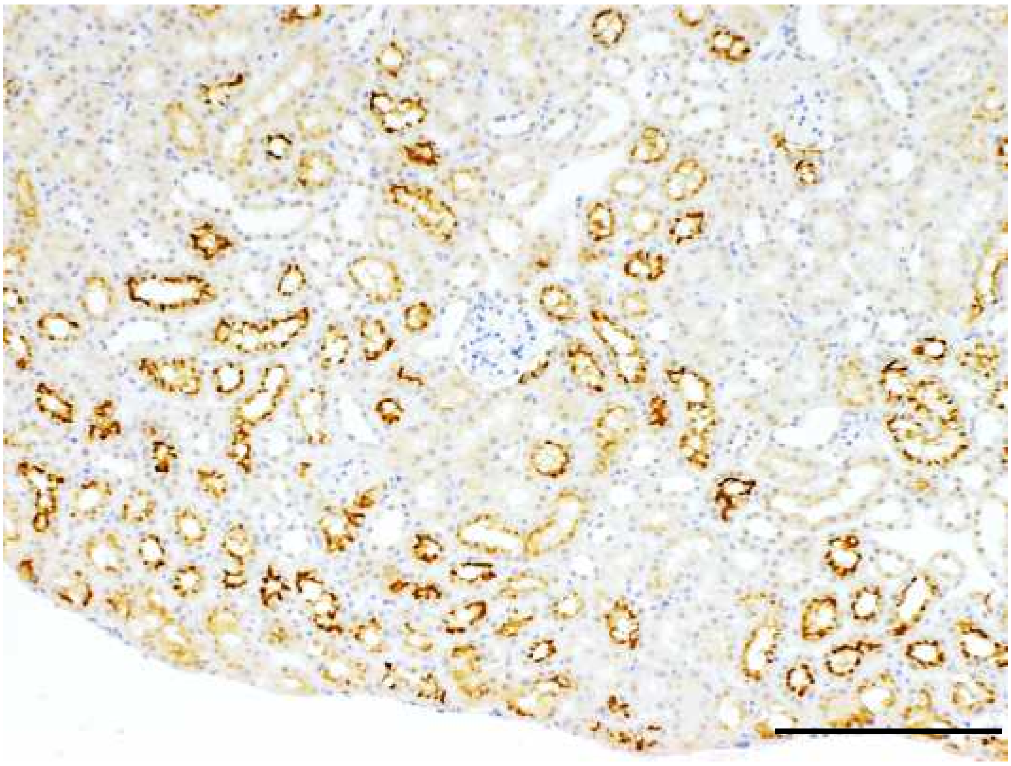

HPF

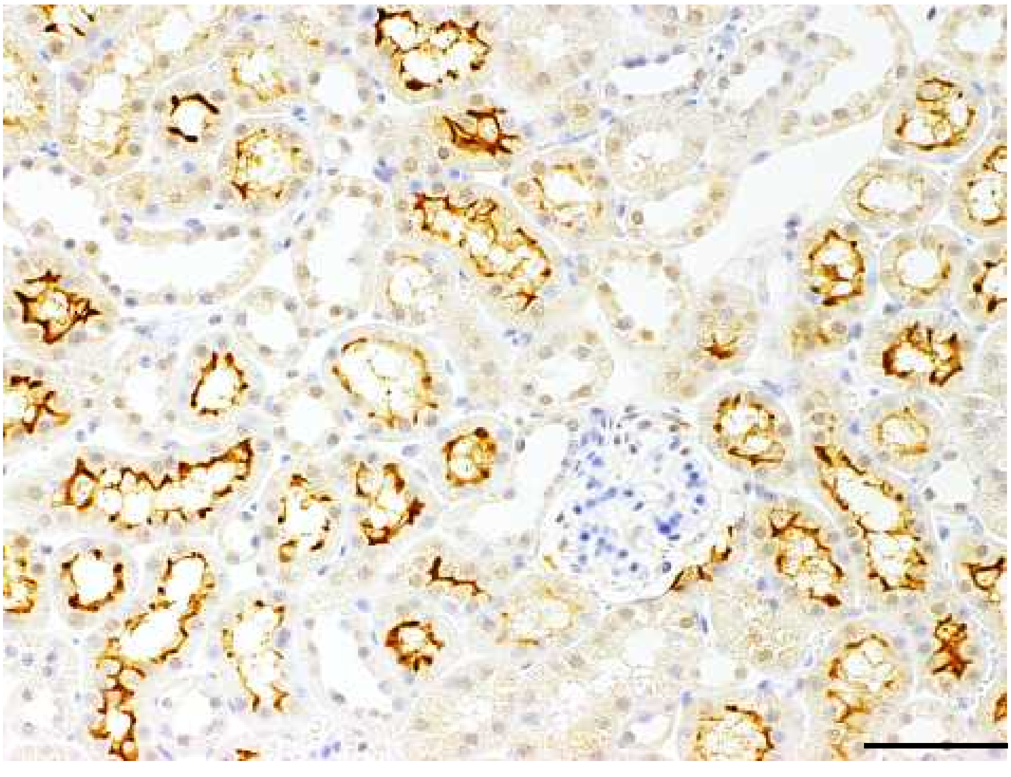

Supplementary figure 8

Sglt2 staining (low and high magnification)

db/db low Cana

LPF

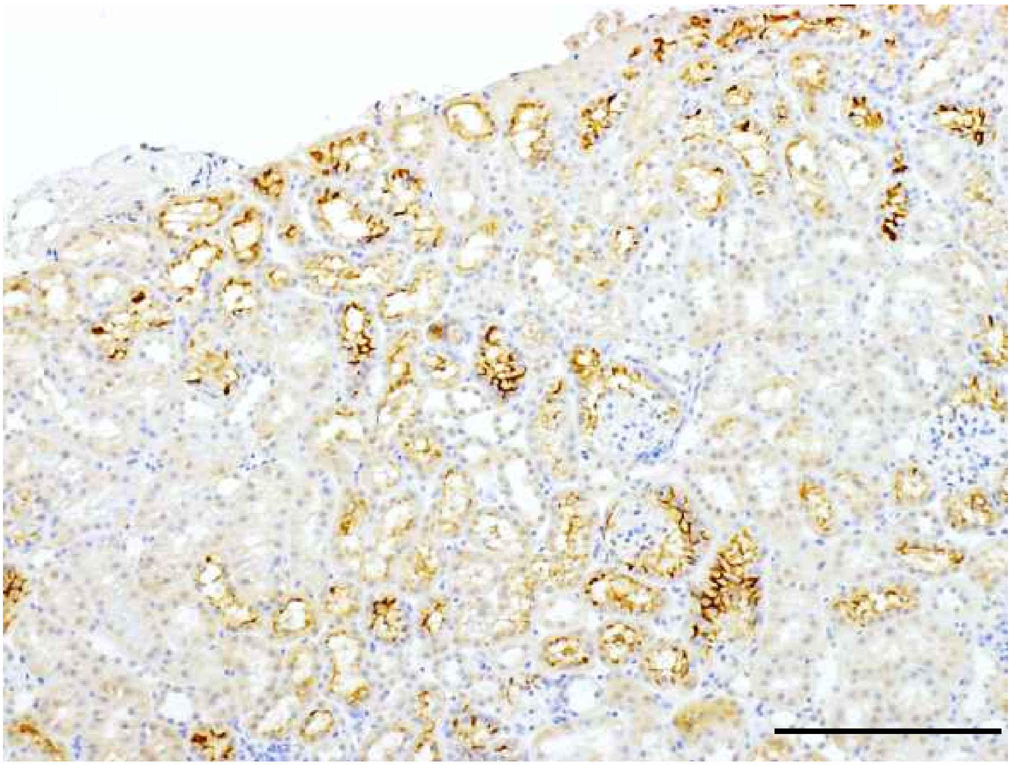

HPF

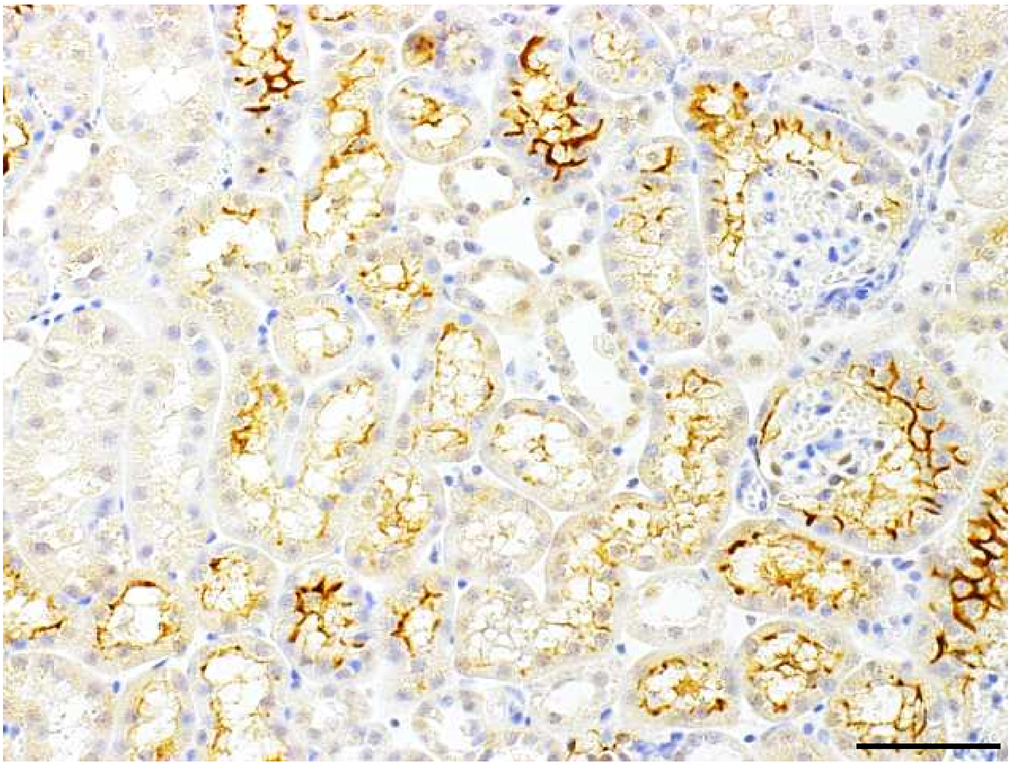

Supplementary figure 9

Sglt2 staining (low and high magnification)

db/db high Cana

LPF

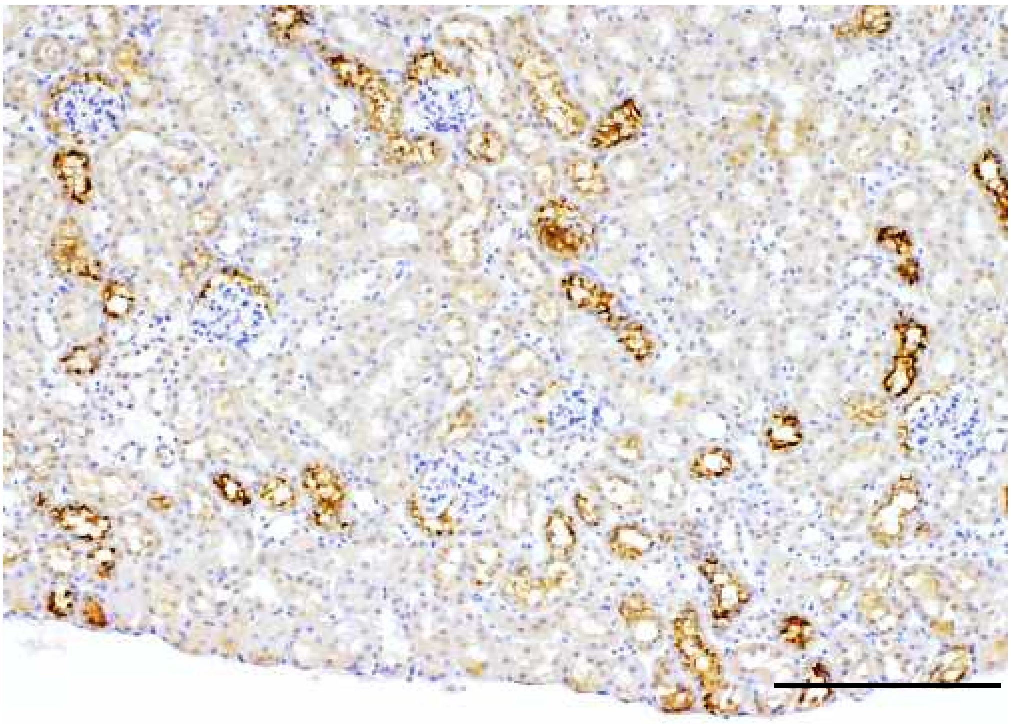

HPF

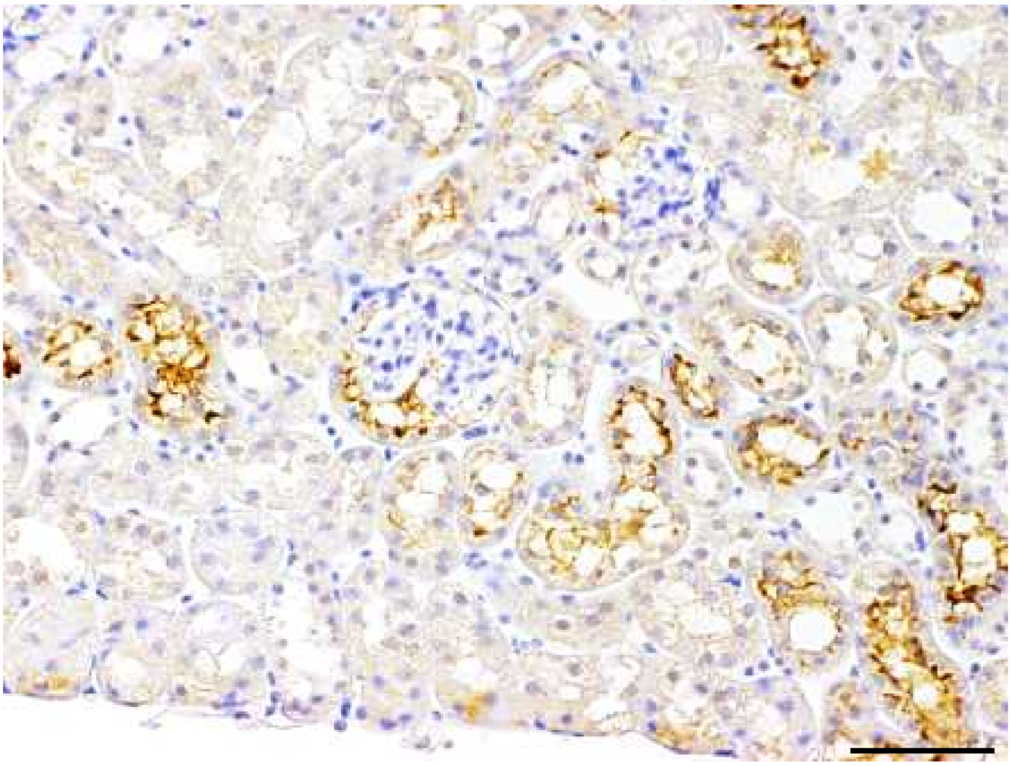

Supplementary figure 10

A

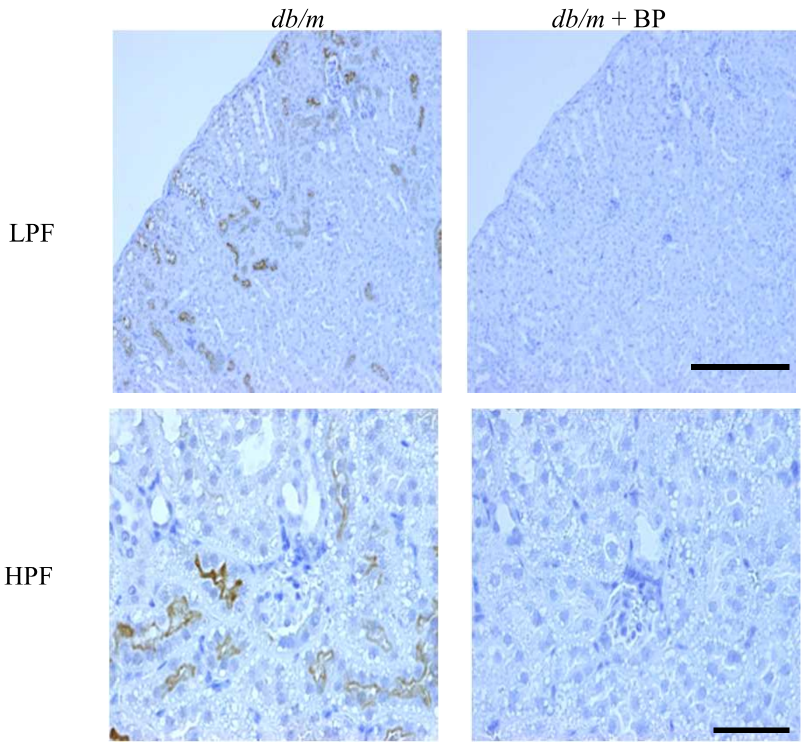

B

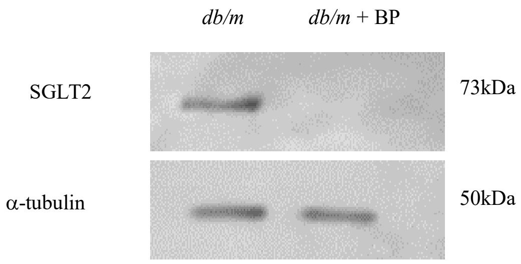

Supplementary figure 11

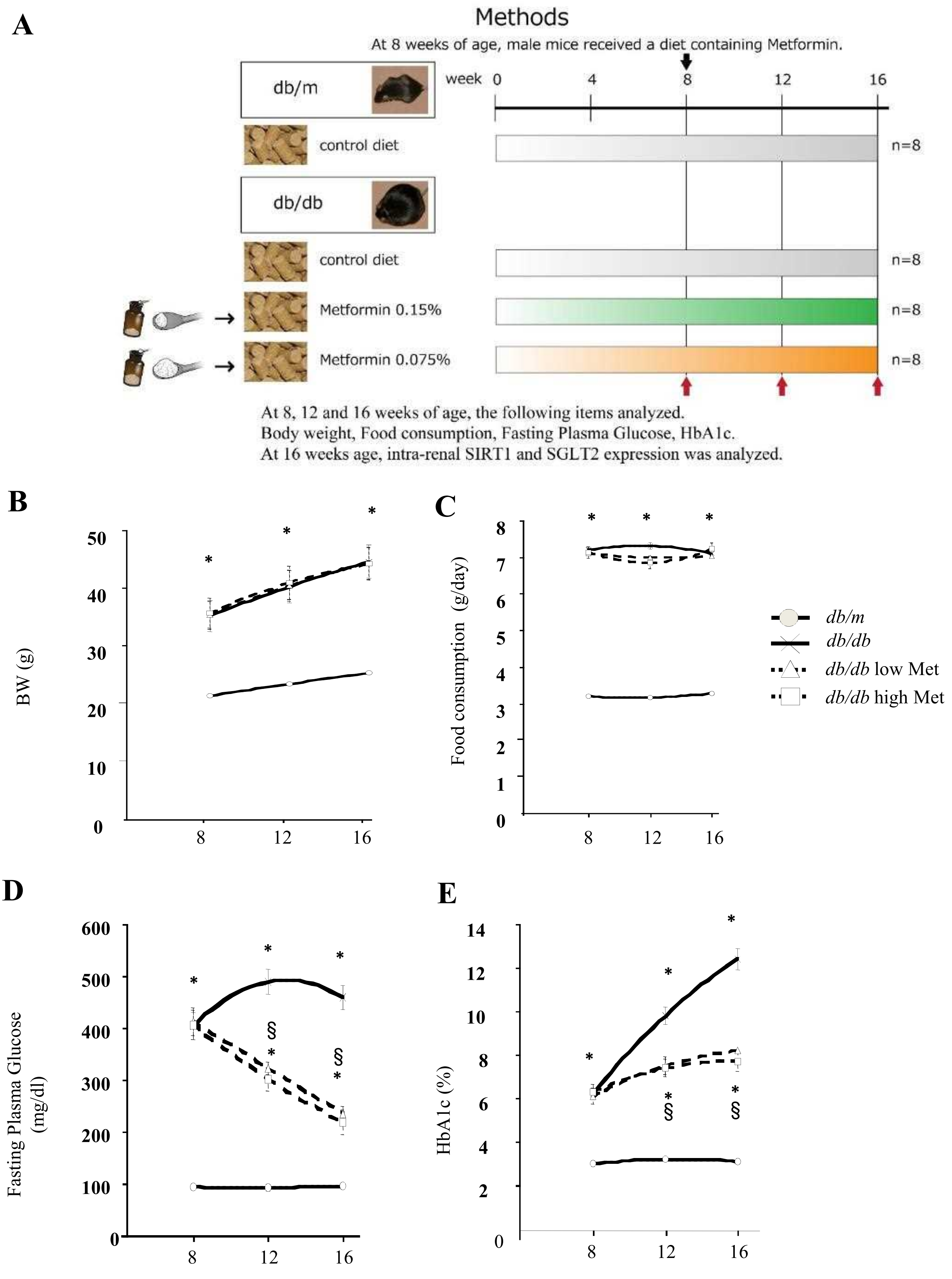

Supplementary figure 12

Metformin

A

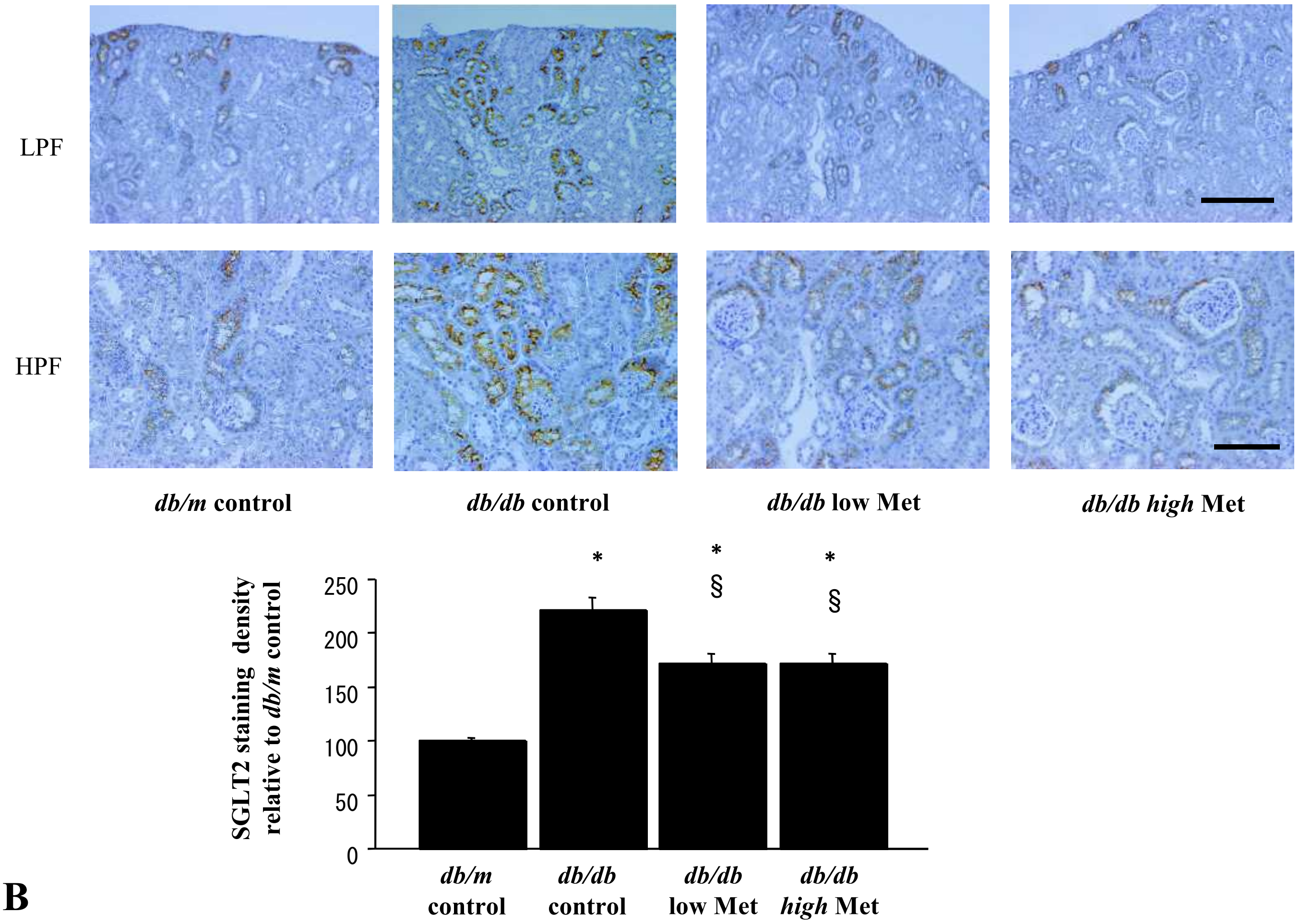

B

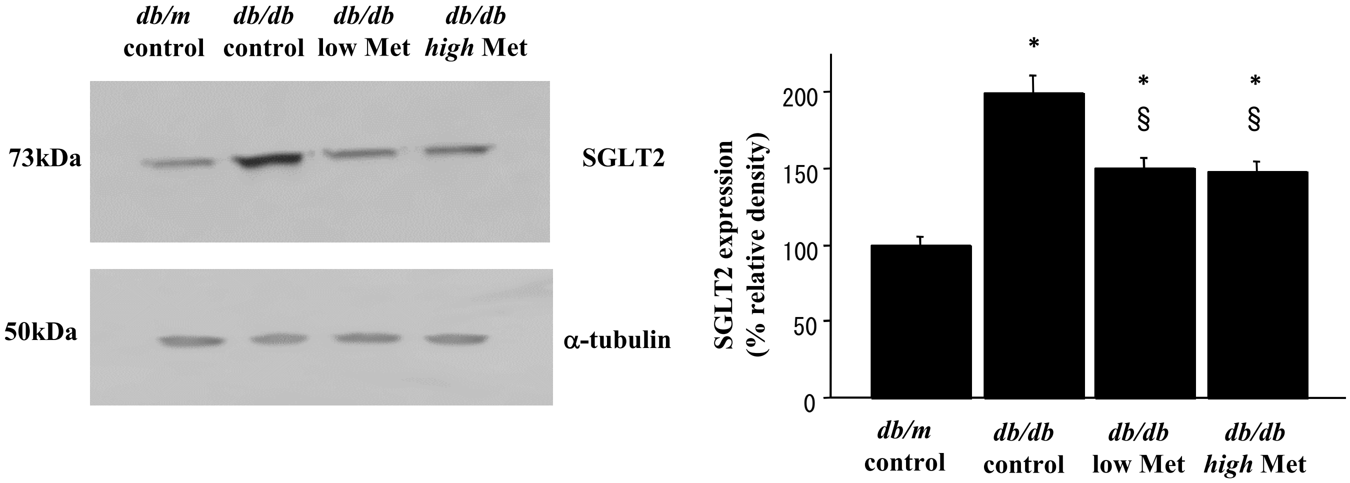

Supplementary figure 13

A

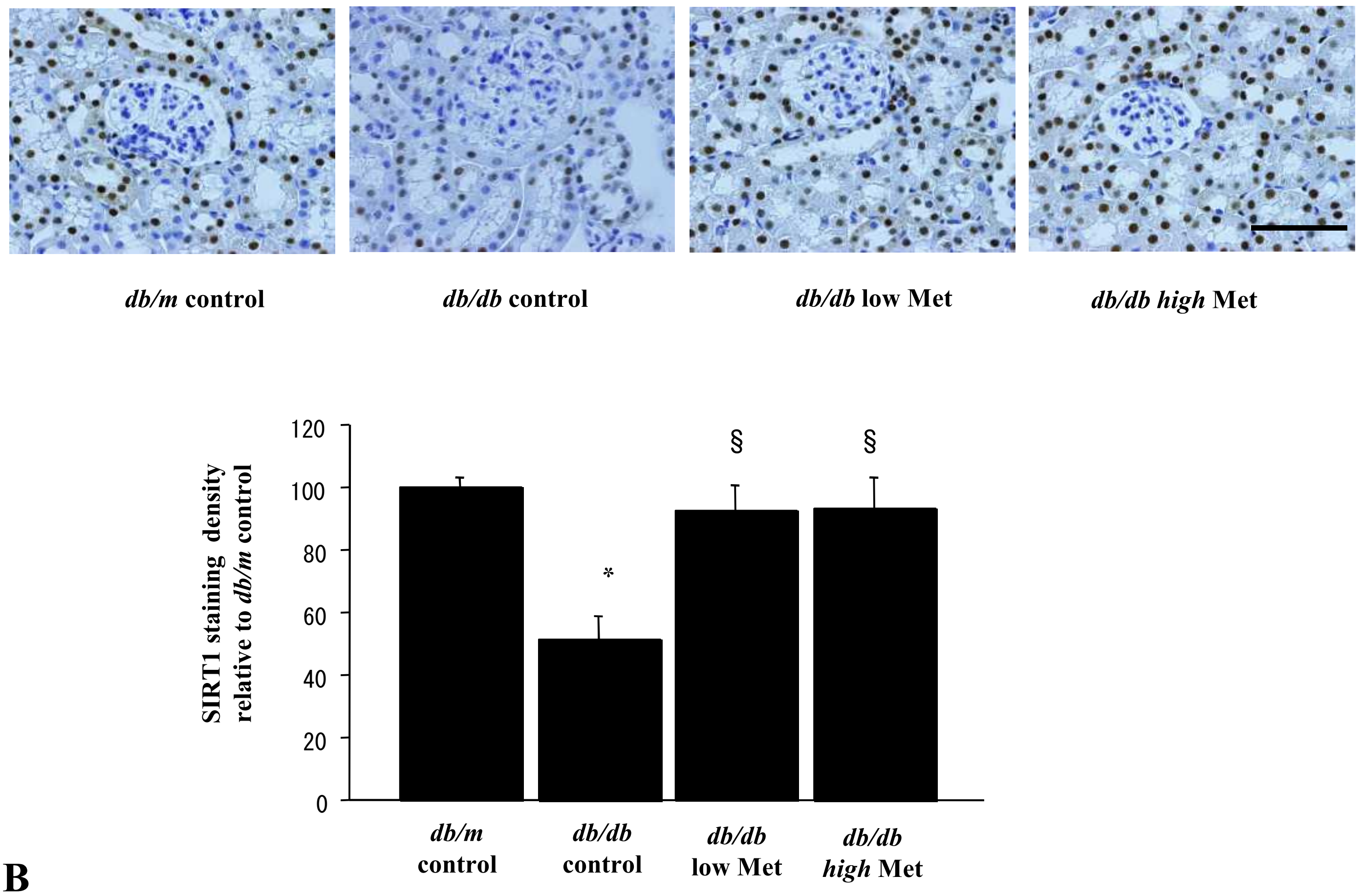

B

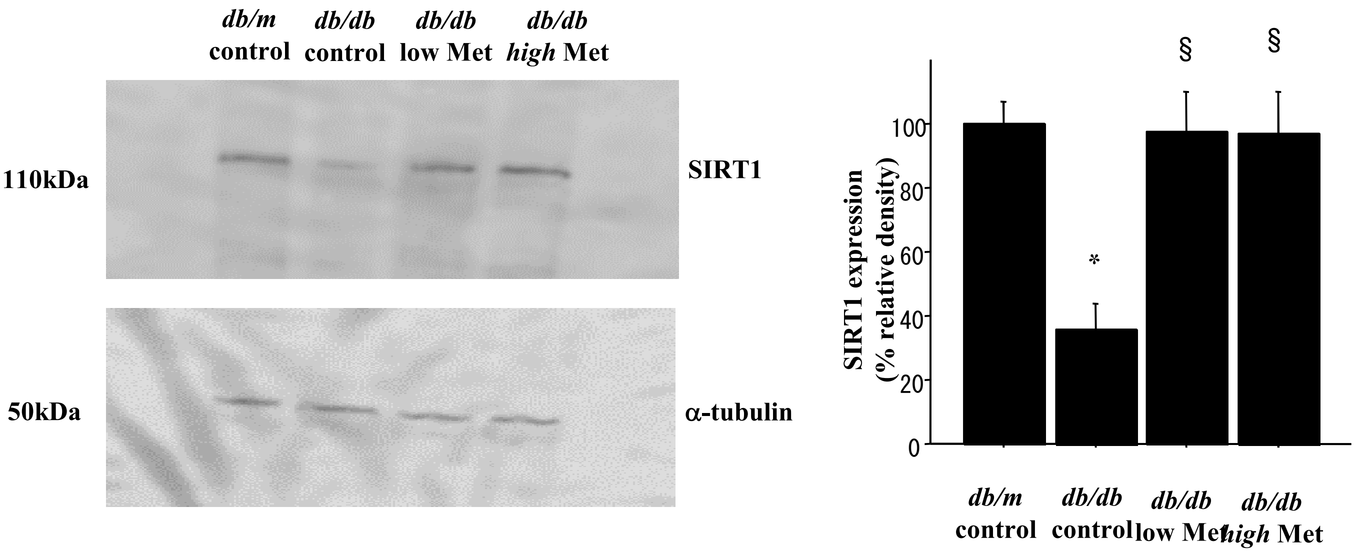

Supplementary figure 14

A

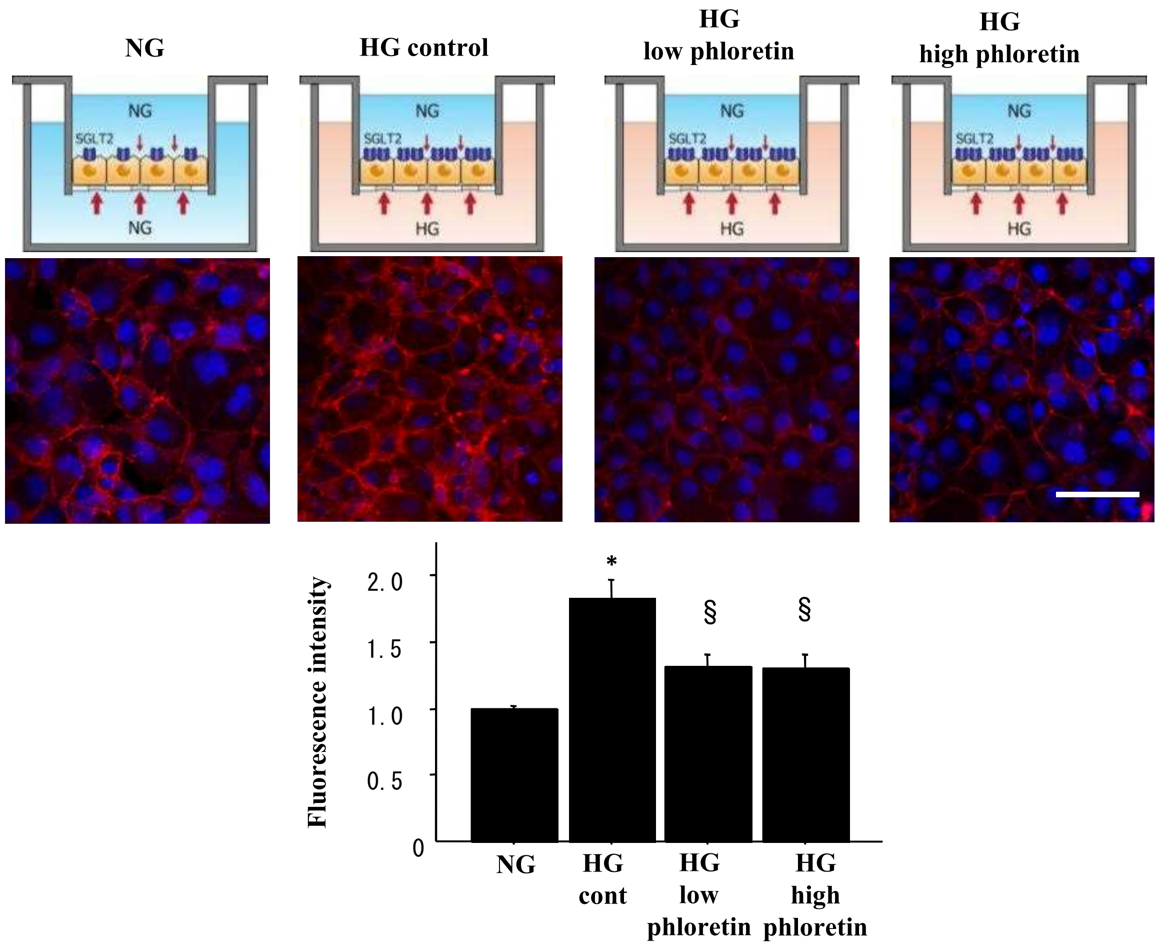

B

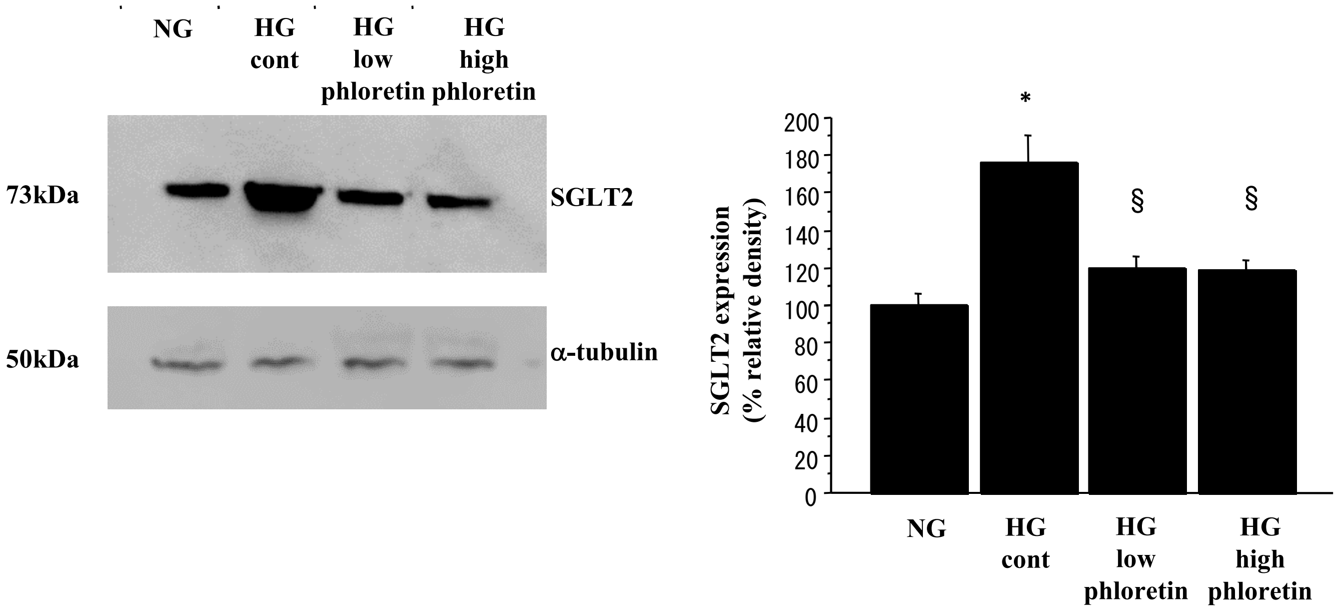

Supplementary figure 15

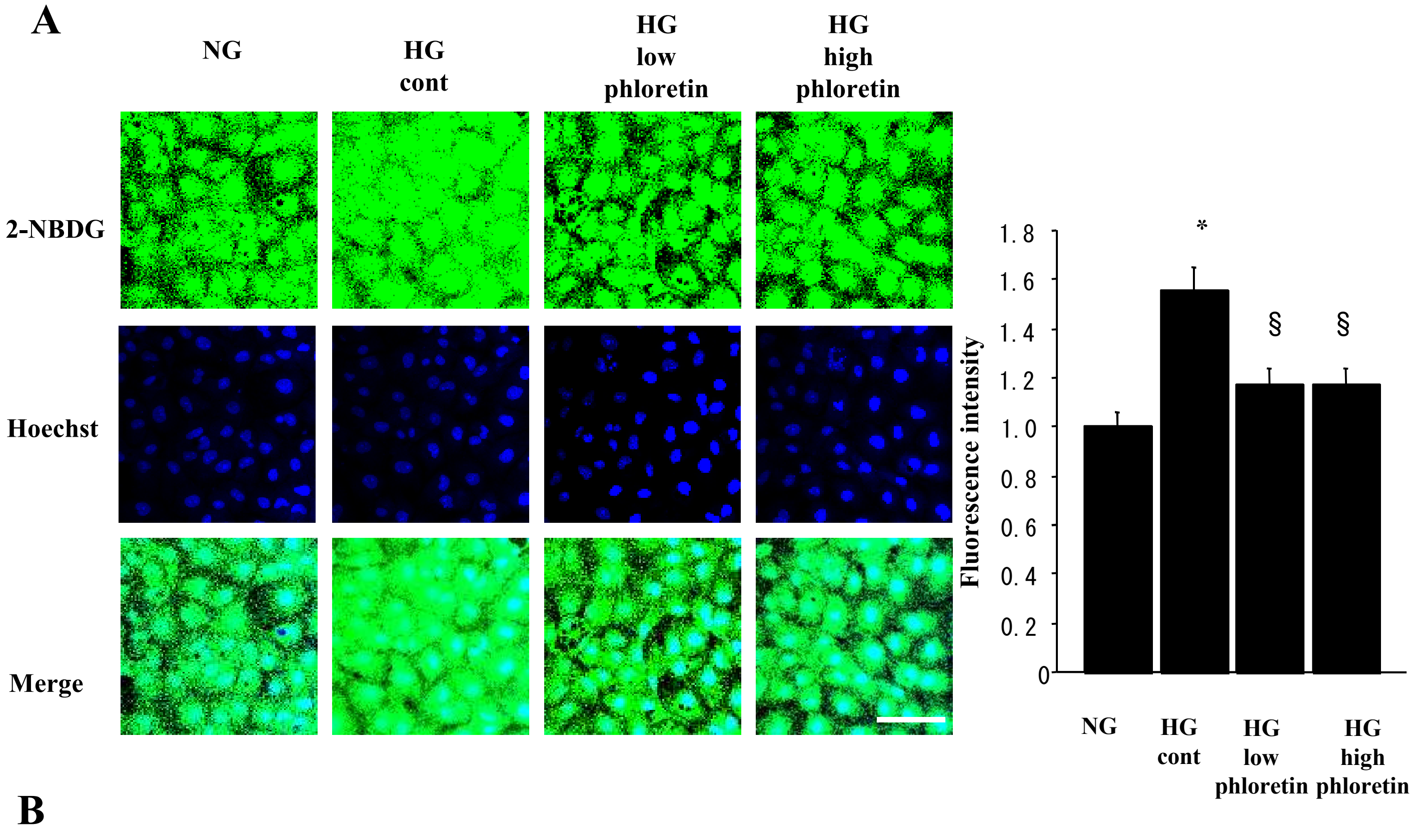

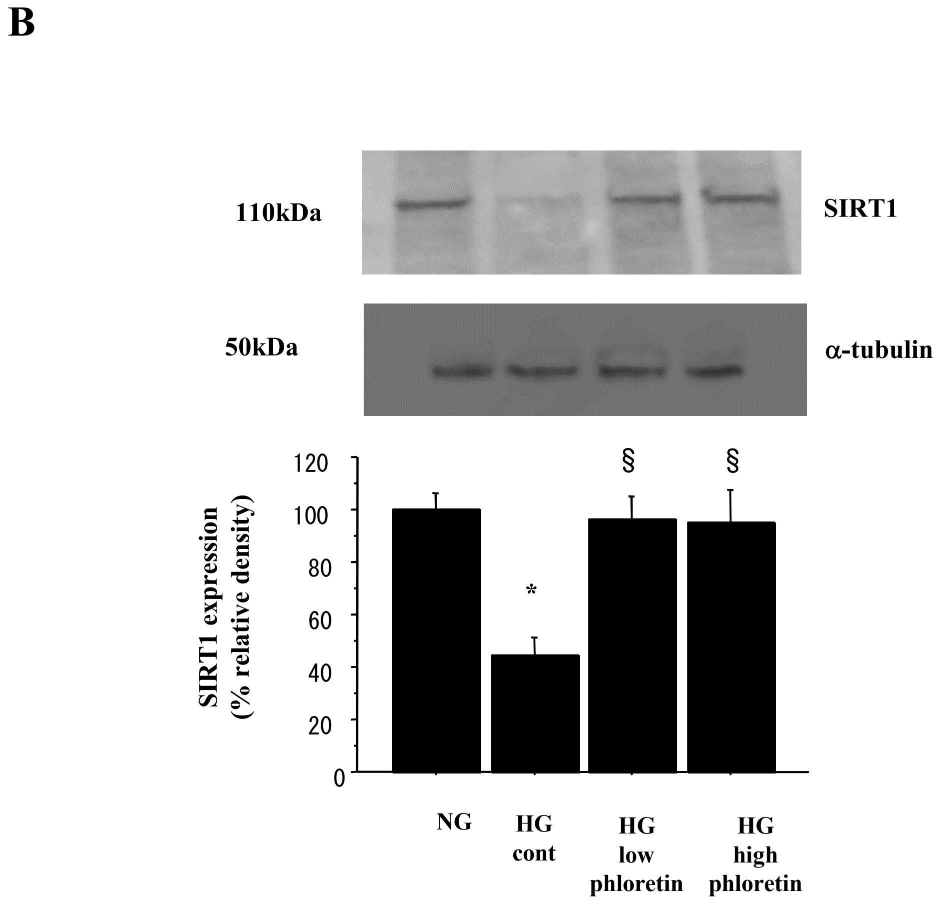

Supplementary figure 16

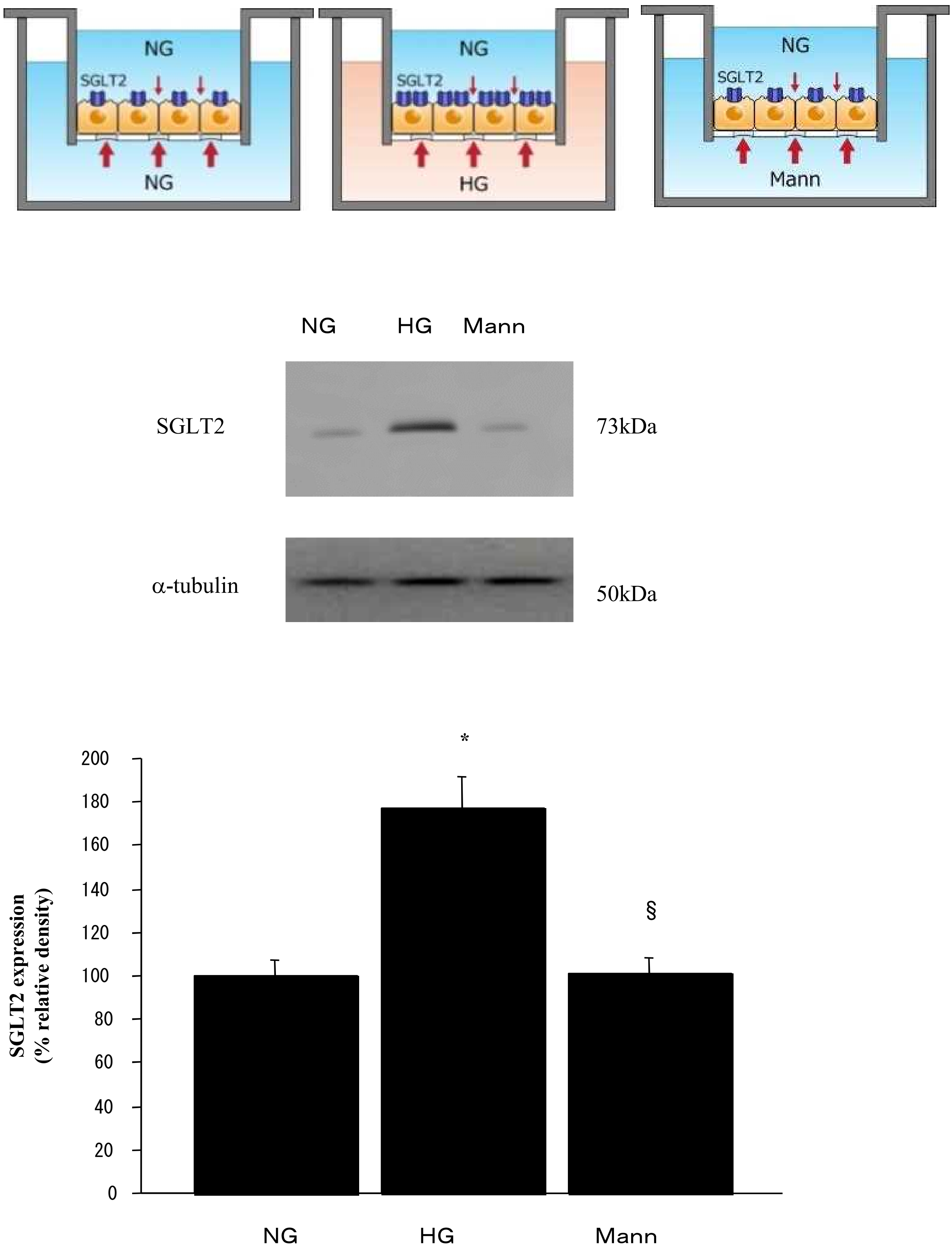

Supplementary figure 17

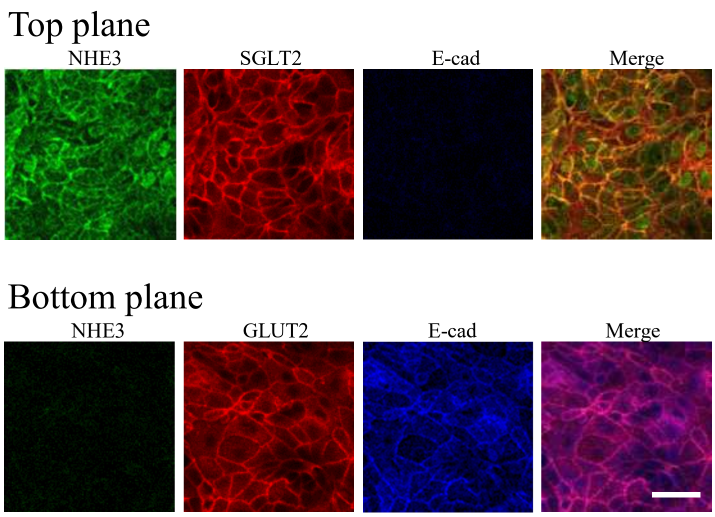

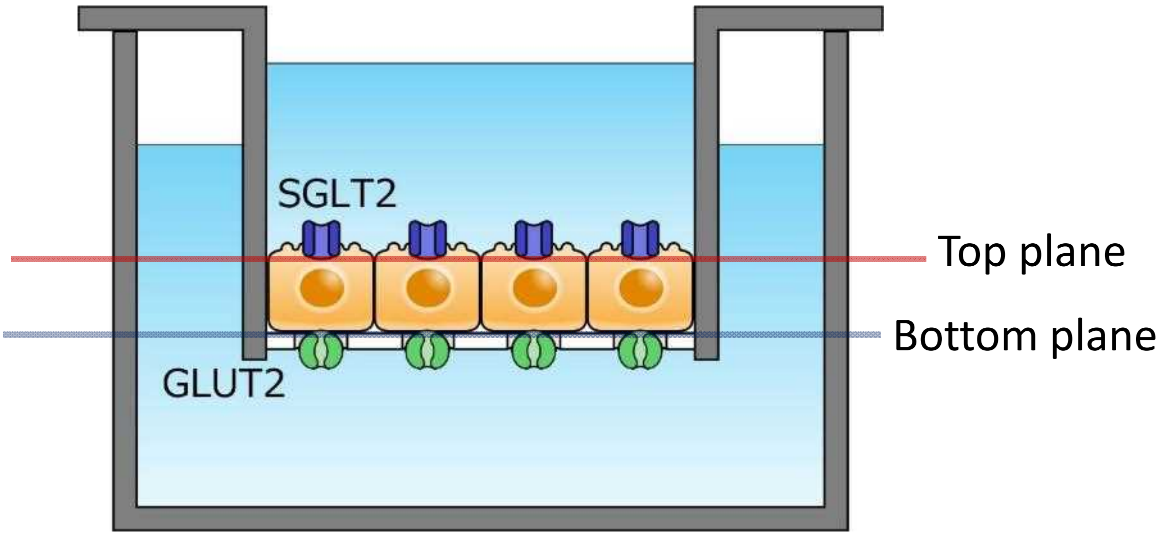

Supplementary figure 18

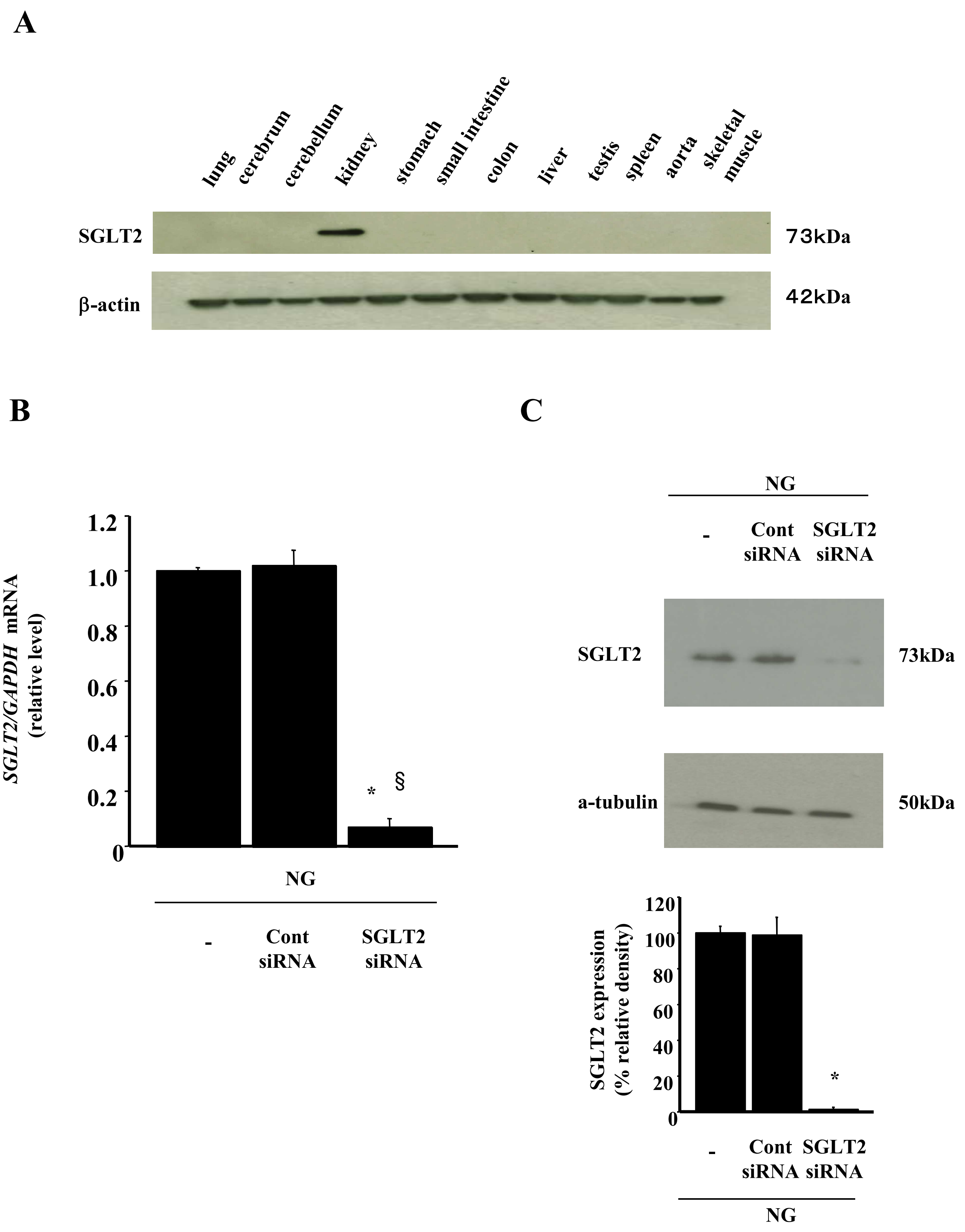

Supplementary figure 19

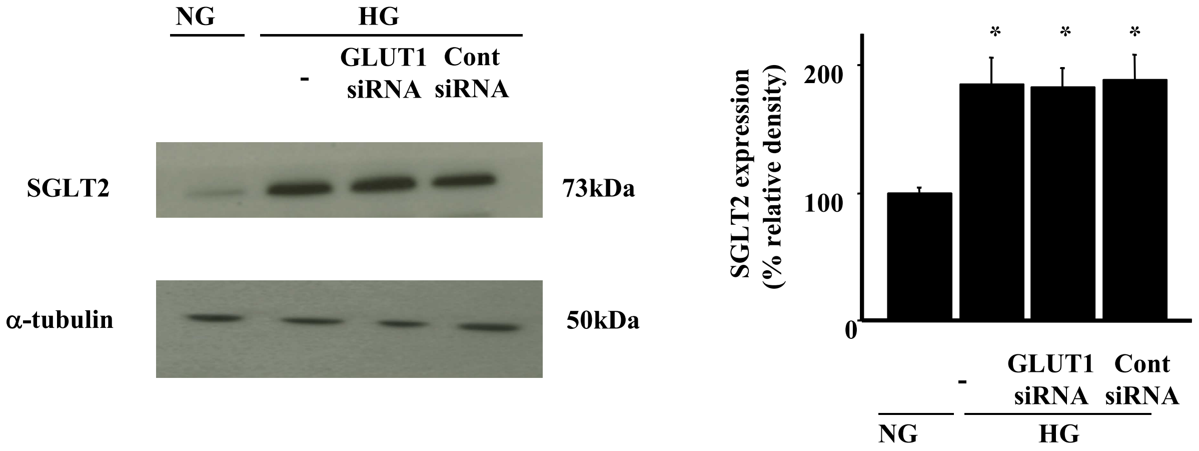

Supplementary figure 20

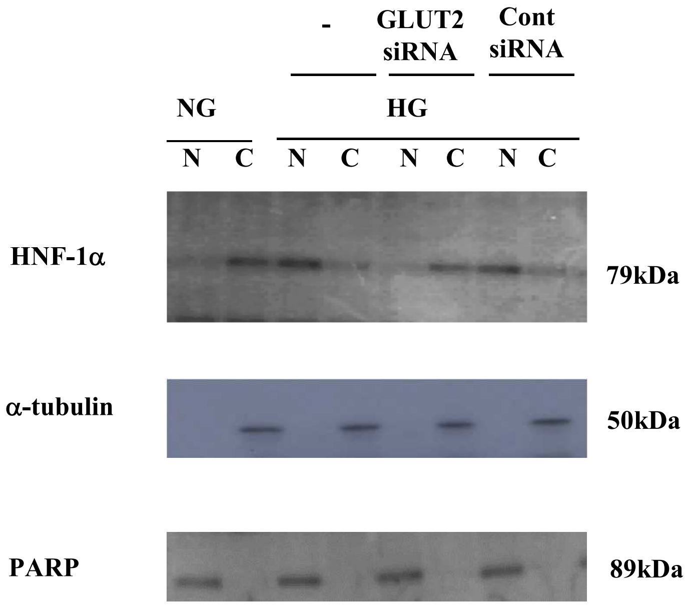

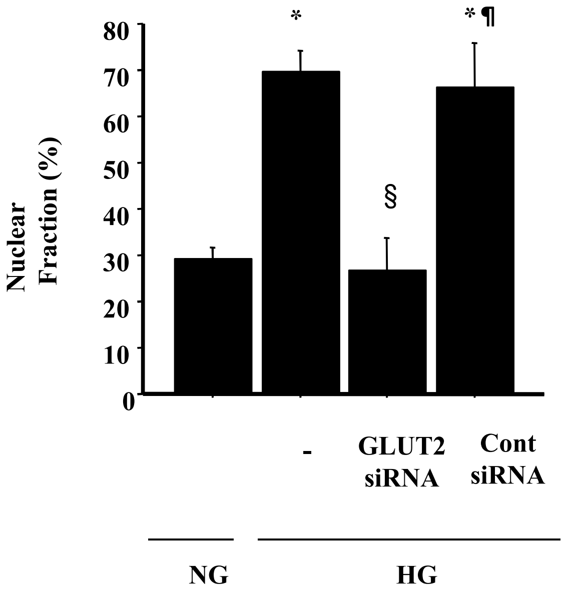

Supplementary figure 21
